# Health risk assessment of Sudan dyes, toxic elements, and pesticide residues in Egyptian spices

**DOI:** 10.1038/s41598-025-31386-3

**Published:** 2025-12-23

**Authors:** Mahmoud M. Ghuniem, Hoda M. Refai, Khaled Rabie

**Affiliations:** Ministry of Agriculture and Land Reclamation, Agricultural Research Center, Central Laboratory of Residue Analysis of Pesticides and Heavy Metals in Foods (QCAP Egypt), 7-Nadi El-said Street, Dokki, P.O. 12311, Giza, Egypt

**Keywords:** Environmental contaminants, Heavy metals, Sudan dyes, Pesticides, Hazard quotient, hazard index, spices, Metals, Plant sciences, Health care, Chemistry

## Abstract

Sudan dyes, toxic elements, and pesticide residues in spices pose potential health risks through long-term exposure, emphasizing the need for regular monitoring. This study evaluated their occurrence and toxicological impact in 80 spice samples collected from Cairo, Giza, Qalyubia, Faiyum, and Alexandria governorates in Egypt. Advanced analytical techniques including High-Performance Liquid Chromatography (HPLC), Gel Permeation Chromatography (GPC), Inductively Coupled Plasma Mass Spectrometry (ICP-MS), Liquid Chromatography Tandem Mass Spectrometry (LC-MS/MS), and Gas Chromatography-Mass Spectrometry (GC-MS) were used for detection. Several metals: antimony (Sb), arsenic (As), cadmium (Cd), chromium (Cr), cobalt (Co), copper (Cu), iron (Fe), lead (Pb), manganese (Mn), nickel (Ni), and zinc (Zn) were found in nearly all samples, except As, which appeared in 75%. Mercury (Hg) and tin (Sn) were below quantification limits. Lead exceeded permissible limits in 5% of hot chili, paprika, and cumin samples. Despite being banned, Sudan I and IV dyes were detected in all tested commodities, reaching 52.3 mg/kg in curry and 6.5 mg/kg in hot chili, while Para Red and Sudan 7B were absent. Although metals and pesticides were within safe exposure limits, the detection of Sudan dyes indicates potential health concerns and the need for continued regulatory control.

**Supplementary Information:**

The online version contains supplementary material available at 10.1038/s41598-025-31386-3.

## Introduction

Spices, derived from the dried parts of plants, have long been recognized for their ability to enhance the sensory properties of foods by imparting desirable aroma, color, and flavor, while occasionally masking undesirable odors^1,2^. Various plant components such as seeds, fruits, roots, and barks serve as key ingredients in cooking, playing a vital role in both nutrition and the cultural development of societies worldwide. Although fresh spices such as ginger generally provide more intense flavors, they are often costlier and have a shorter shelf life than their dried counterparts. Moreover, fresh forms are not always readily available throughout the year.

Although many health benefits are attributed to spices, current scientific evidence supporting these claims remains limited. India dominates global spice production, accounting for approximately 75% of the total output^3,4^. Beyond their sensory appeal, spices are also valued for their diverse applications in traditional medicine, religious practices, cosmetics, and perfumery^5^. These versatile ingredients are derived from various plant parts, including rhizomes, barks, leaves, fruits, and seeds.

Historically, spices have significantly influenced human civilization, trade, and exploration due to their dual role as food condiments and remedies for illnesses^6,7^. Over the past three decades, their usage has grown substantially worldwide, largely driven by their perceived medicinal properties. While spices are naturally cultivated in many regions, they are also grown on small farms^8^. Among the most exported spices are ginger, chilies, turmeric, and black cumin. Studies reveal that spices such as ajwain, black cumin, coriander, and fenugreek collectively account for about 36% of the cultivated area and 17% of the total spice production in certain regions^9^.

Heavy metals can enter the food chain through crop mineralization or environmental contamination, such as the use of agricultural inputs like pesticides, fertilizers, or irrigation with polluted water sources^10^. Some heavy metals, including chromium, copper, iron, manganese, and zinc, are essential for humans and play critical roles in various biochemical processes^11^. However, excessive intake of these metals can lead to toxicity. In contrast, non-essential heavy metals such as lead, cadmium, mercury, and arsenic are toxic even at low concentrations^12,13^.

Regular consumption of food contaminated with heavy metals can result in their accumulation in human organs, posing significant health risks over the short, medium, and long term^14^. Consequently, regular monitoring of heavy metal contaminants is recommended to mitigate these risks^15^.

Regarding spice safety, limited information is available in the literature on heavy metal contamination. For instance, lead (Pb) levels exceeding permissible limits have been reported in unbranded turmeric samples^16^. Other studies have analyzed heavy metal concentrations in spices and found that most were within permitted levels, deeming them safe for human consumption^17,18^.

Edible colorants are a vital category of food additives, divided into natural and synthetic types^19^. Certain synthetic colorants, particularly azo dyes, are restricted or banned because they can be easily reduced under anaerobic conditions to form aromatic amines, which pose health risks^20^. Sudan dyes, a class of azo dyes, are prohibited in food products but are still found in various foods^21^. These dyes are widely used in industrial and scientific applications, such as fuel staining and coloring, due to their low cost and availability. Despite their appeal as food colorants, their usage is strictly regulated.

Sudan dyes must not exceed the maximum limits set by Commission Decision No. 2011/1129/E^22^, and Egyptian authorities prohibit the import of products containing unauthorized dyes, even if permitted in other countries^23^. Sudan I, II, III, and IV have been classified as Group 3 carcinogens by the International Agency for Research on Cancer (IARC). To enhance market supervision and protect public health, these dyes are banned under Commission Decisions No. 2003/460/EC and 2004/92/EC^24^.

Pesticides are classified based on their toxicity, the range of pests they target, their function, chemical composition, mechanism of entry, and mode of action. In the 19th century, pesticides such as pyrethrums derived from chrysanthemums and rotenone from tropical plant roots were introduced. Until the 1950 s, arsenic-based pesticides were widely used^25^. Later, organochlorines like DDT gained popularity but were phased out in the U.S. by 1975 and replaced by organophosphates and carbamates. By the mid-1990s, DDT was banned in over 86 countries due to its environmental and health risks. Since then, pyrethrin-based pesticides have become the most widely utilized. Herbicides gained prominence in the 1960 s, particularly nitrogen-based compounds like triazine, carboxylic acids such as 2,4-dichlorophenoxyacetic acid, and glyphosate^26^.

Over the past 60 years, pesticides have significantly enhanced food production by reducing pest-related agricultural losses and ensuring year-round availability of food at affordable costs. Their use has notably increased agricultural yields in countries like the UK, US, and Russia^27,28^. However, pesticides also contaminate ecosystems, entering seaweeds and microorganisms and accumulating in fish, leading to human exposure through seafood consumption^29^.

Exposure to pesticides poses short-term acute health risks, including nausea, vomiting, skin and eye irritation, sores, headaches, dysentery, and even death. Long-term exposure, particularly in agricultural workers, can lead to severe respiratory issues such as chronic cough, asthma, reduced lung function, bronchitis, and other pulmonary conditions^30,31^.

Despite the widespread consumption of spices in Egypt, limited comprehensive data exist regarding their contamination with Sudan dyes, toxic elements, and pesticide residues, particularly considering their combined occurrence and associated health implications. Previous studies have often focused on one or two contaminant groups, overlooking their potential cumulative risk. Therefore, this study aimed to provide a holistic evaluation of the occurrence and toxicological impact of these contaminants in commonly consumed spices. Specifically, it assessed the presence of seven Sudan dyes, thirteen potentially toxic elements, and 461 pesticide residues in eighty spice samples collected from Giza governorate, Egypt. To the best of our knowledge, this is the first study in Egypt to integrate multi-residue analysis using advanced chromatographic and spectrometric techniques (HPLC, GPC, ICP-MS, LC-MS/MS, and GC-MS) alongside comprehensive dietary exposure and health risk assessment. This approach provides novel insights into the co-occurrence and potential health risks of multiple contaminant classes in spices marketed locally.

## Materials and methods

### Instruments

For the analysis of heavy metals, an Ethos Up High-Pressure Microwave System from Milestone (Italy) was employed. This system was coupled with a Perkin Elmer NexION 2000 Inductively Coupled Plasma Mass Spectrometer (USA), equipped with components including an autosampler S10, SV40 BI vacuum pump, copper coil RF, skimmer cone, sampler cone, hyper skimmer cone, Meinhard concentric glass nebulizer (C 0.5), ion lens, mist cyclonic spray chamber, quartz torch, and chiller.

For the determination of Sudan dyes, a GPC (Gel Permeation Chromatography) clean-up system from Waters (USA) was used. This system included a built-in binary pump (Waters 1525), a 100-vial autosampler tray (model Waters 717 Plus) with a 5000 µL sample loop, and a fraction collector equipped with Bio-Beads SX3 (200–400 mesh) from Bio-Rad for GPC column processing. Detection was performed using a High-Performance Liquid Chromatography (HPLC) system, model HP Agilent 1200 series (Germany), featuring a vacuum degasser (G1379B), fluorescence detector (Agilent 1260 infinity/1200 series G1321A), quaternary pump (G1311A), autosampler (G1313A), and an analytical column (Agilent Eclipse Plus C18, 5 μm, 4.6 × 250 mm). The system operated with ChemStation software (Rev. B.04.03).

For pesticide residues, an HP (Agilent) 1100 series High-Performance Liquid Chromatography system equipped with a binary pump, vacuum degasser, and autosampler was utilized. Nitrogen gas was supplied at a minimum flow rate of 5 L/min at 60 psi, source gas (zero air) at 22 L/min at 100 psi, and exhaust gas (air) at 8 L/min at 60 psi. LC-MS/MS analysis was performed using an API 4000 Q-Trap (Applied Biosystems) triple quadrupole mass spectrometer with a mass range of m/z 50–2800. This system featured a Turbo V ion source capable of electro-spray ionization (Turbo-Ion Spray) and atmospheric pressure chemical ionization (ESI/APCI) in both positive and negative modes.

Additionally, a Gas Chromatography system (Agilent 7890 A) coupled with a 7000B series tandem mass spectrometer (GC-MS/MS) was employed. This system included a triple quadrupole GC/MS EI mainframe with femtogram-level limits of detection, an ultra-low noise ion gauge controller, and a splitless inlet. The carrier gas used was helium, with a flow rate of 1.830 mL/min. The analytical column was an HP-5 MS capillary column (Agilent Technologies) with a 5% biphenyl and 95% dimethyl siloxane stationary phase, an internal diameter of 0.25 mm, a film thickness of 0.52 μm, and a length of 30 m.

#### Apparatus

The study utilized a range of advanced laboratory equipment to ensure precise sample preparation and analysis. A Heidolph Rotary Evaporator was employed for solvent evaporation, and a Sigma Centrifuge with speeds up to 4500 rpm was used for sample separation. A Geno/Grinder 2010 SPEX Sample Shaker facilitated efficient sample homogenization. For accurate volume measurements, calibrated micropipettes (10–100 µL and 100–1000 µL ranges) from Hirschman Laborgeräte (Germany) were utilized.

For water purification, a Millipore Water Purification System equipped with a Q-POD element and coupled with a Merck Millipore-Q^®^ Integral 5 (A10^®^) system provided high-purity water. Solvents were dispensed with a 10 mL solvent dispenser (Hirschman Laborgeräte, Germany). Additionally, a Mettler Toledo Top Bench Balance with a measurement range from 0.1 mg to 210 g was used for precise weighing tasks.

### Chemicals and reagents

For the analysis of heavy metals, Suprapur^®^ nitric acid (HNO_3_) (65% weight/weight) from Merck (Germany) was used, along with Emsure^®^ Hydrogen Peroxide (H_2_O_2_) at 30% concentration, also from Merck (Germany). The deionization of water was carried out in-house using a Millipore water purification system. A 2% volume/volume nitric acid solution was prepared following the procedure described in^12^.

For the analysis of Sudan dyes, acetonitrile, ethyl acetate, and n-hexane (all HPLC grade) were sourced from Sigma-Aldrich (Germany), while Bio-Beads SX3 (200–400 mesh) from Bio-Rad and sodium sulfate were also used.

High-purity solvents and reagents were used for pesticide residue analysis. n-Hexane, toluene, acetonitrile, methanol, and tetradecane were sourced from Sigma-Aldrich, Merck, and Aldrich. QuEChERS extraction salts were obtained pre-mixed from Agilent Technologies, and deionized water was produced using a Millipore system. Several solutions were freshly prepared, including 10 M NaOH, 1 M citric acid (pH 4.0), 10% ammonium hydroxide, and 0.5 M Na2-EDTA, following standard laboratory procedures for dissolution and pH adjustment. Formic acid (98–100.5%) was supplied by Riedel-de Haën. Sample cleanup was performed using Oasis MCX solid-phase extraction cartridges (Waters) following the procedure described in^32,33^.

### Certified reference material

For heavy metal analysis, stock standard solutions (1000 mg/L in 2–3% HNO₃) of As, Pb, Cd, Sb, Hg, Cu, Zn, Fe, Cr, Sn, Co, Mn, and Ni were obtained from Merck (Germany). A certified NexION setup standard containing Be, Ce, Fe, In, Li, Mg, Pb, and U at 1 µg/L in 1% HNO₃, as well as a certified internal standard mixture comprising Bi, Ge, In, ⁶Li, Sc, Tb, and Y at 10 µg/mL in 5% HNO₃, were procured from PerkinElmer (USA).

For the determination of Sudan dyes, analytical standards of Orange G, Para Red, Sudan I, Sudan II, Sudan III, Sudan Red 7B, and Sudan IV were purchased from LGC.

For pesticide residue analysis, reference standards for approximately 461 pesticides (purity > 95%) were sourced from Dr. Ehrenstorfer (Augsburg, Germany). Stock solutions (1000 µg/mL) were prepared in toluene and stored at − 20 ± 2 °C. These standards were used for LC–MS/MS and GC–MS/MS analyses. A solvent system consisting of n-hexane and acetone (9:1, v/v) was employed owing to its favorable solubility, stability, and compatibility with the targeted pesticides.

### Standards Preparation

#### Heavy metals

Following the procedures described in^12^, eight working standard solutions were prepared for As, Pb, Cd, Sb, and Hg within a concentration range of 0.05–100 µg/L. For Fe, Sn, Cu, and Zn, nine working standards were prepared covering 1–5000 µg/L. Additionally, ten working standards were prepared for Mn, Cr, Co, and Ni ranging from 0.05 to 1000 µg/L.

#### Sudan dyes

In accordance with the methodology outlined in^23^, individual stock, intermediate, and working standard solutions (1000 mg/kg) were prepared for each Sudan dye in a mixture of acetonitrile and dichloromethane (1:1, v/v). A combined 100 mg/kg spiking mixture was subsequently prepared in the same solvent system. Calibration standards for HPLC were also prepared using the same solvent mixture. Stock, intermediate, and working standards were stored at − 20 °C, while calibration and spiking mixtures were maintained at 4 °C until analysis.

#### Pesticide residues

Following the preparation procedures described in^32,33^, stock solutions (1000 µg/mL) were prepared individually for each pesticide in an appropriate solvent. Intermediate mixtures (10 µg/mL) were prepared separately for LC-amenable and GC-amenable pesticides in toluene. Both mixtures were further diluted to obtain a 2.5 µg/mL spiking mixture in toluene. For GC-MS/MS, a 100 µg/mL aldrin solution in n-hexane was used as an internal standard. Calibration solutions for GC-MS/MS (0.01, 0.05, 0.1, 0.5 µg/mL) were prepared in blank green bean extract obtained by the QuEChERS method, using a solvent system of n-hexane/acetone (9:1, v/v) and supplemented with 0.1 µg/mL aldrin. LC-MS/MS calibration standards (0.005–0.5 µg/mL) were prepared in methanol. Stock, intermediate, and working solutions were stored at − 20 °C, whereas calibration and spiking mixtures were stored at 4 °C prior to analysis.

### Sample collection

According to the sampling procedures outlined by the Egyptian Standards and the Codex Alimentarius Commission^34–36^, a total of 80 samples of hot chilli, paprika, cumin, and curry were randomly collected from the Cairo, Giza, Qalyubia, Faiyum, and Alexandria governorates. The collected samples were unprocessed and stored in plastic containers. Each sample was labeled with an identification code to ensure traceability. The samples were then stored at 4 °C until further analysis.

### Sample Preparation

#### Heavy metal analysis

Following the protocol described in^12^, spice samples were homogenized, and 0.5 g was transferred into microwave digestion vessels. Each vessel received 8 mL of Suprapur nitric acid and was gently shaken before adding 2 mL of hydrogen peroxide. Vessels were sealed and digested in a microwave system equipped with a thermocouple probe inserted in a reference vessel for temperature monitoring. The digestion program operated at 1800 W for 15 min, increasing the temperature to 200 °C, which was maintained for an additional 15 min. Vessels were allowed to cool below 80 °C before opening. Digestates were rinsed with deionized water and transferred to 50 mL polypropylene tubes. An internal standard mixture (0.5 mL; Bi, Ge, In, ⁶Li, Sc, Tb, Y) was added, and volumes were adjusted with deionized water. A reagent blank was processed identically. Samples were stored in polypropylene tubes until Q-ICP-MS analysis.

#### Sudan dye analysis

As described in^23^, 10 g of sample was extracted with 100 mL acetonitrile by shaking at 200 rpm for 20 min. The extract was filtered through 15 g of activated sodium sulfate, evaporated to dryness, and reconstituted in 4 mL of GPC mobile phase (ethyl acetate: n-hexane, 1:1). One milliliter was injected into the GPC system at 2 mL/min for 24 min. The collected fraction was evaporated to dryness and re-dissolved in 2 mL acetonitrile for HPLC analysis. Chromatographic separation used acetonitrile: water (95:5) at 1 mL/min. Detection wavelengths were 230 nm for all dyes except Sudan Orange G, which was monitored at 380 nm.

#### Pesticide residue analysis

Following^32,33^, 2 g of sample was weighed into centrifuge tubes, hydrated with 10 mL deionized water, and shaken for 1 min. Ten milliliters of acetonitrile was added and mixed for another minute at 4000 rpm. The QuEChERS extraction salts (MgSO₄, NaCl, and citrate buffer) were added, followed by 1 min shaking. Tubes were centrifuged at 4500 rpm for 5 min at 4 °C.

For LC-MS/MS, 1.5 mL of the upper acetonitrile layer was filtered through a PTFE membrane and transferred to an autosampler vial. For GC-MS/MS, 2 mL of extract was evaporated to dryness at 40 °C using a rotary evaporator. Two milliliters of aldrin internal standard (in 9:1 hexane: acetone) was added, ultrasonicated, transferred to a vial, and injected.

Strict contamination-control procedures were implemented throughout sample handling and analysis. Blank solvents and procedural blanks showed no detectable residues. Automated needle-wash cycles with high-purity solvent, disposable sampling tools, and rigorously cleaned laboratory glassware minimized cross-contamination. Swab testing confirmed the cleanliness of work surfaces. These measures ensured that all detected residues originated solely from the analyzed samples.

### Determination

#### Elemental quantification by ICP-MS

Elemental analysis was performed using Inductively Coupled Plasma Mass Spectrometry (ICP-MS) following the procedures described in^12,14^. Isotopes were selected based on natural abundance, minimal spectral interference, and analytical stability, including ⁵⁵Mn, ⁵⁹Co, ⁶⁰Ni, ⁶³Cu, ⁶⁶Zn, ⁵²Cr, ⁵⁶Fe, ⁷⁵As, ¹¹¹Cd, ¹²¹Sb, ¹¹⁸Sn, ²⁰²Hg, and ²⁰⁸Pb. An internal standard mixture (²⁰⁹Bi, ⁷⁴Ge, ¹¹⁵In, ⁶Li, ⁴⁵Sc, ¹⁵⁹Tb, ⁸⁹Y) was added online to all samples and calibration standards to correct for instrumental drift, signal suppression, and matrix effects.

Kinetic Energy Discrimination (KED) with helium as a collision gas was employed to minimize interferences. Helium flow rates were optimized according to element groups: 1.0 mL/min for Pb, Cd, Sb, Sn, Mn, Cu, Co, Ni, and Hg; 4.0 mL/min for Fe and Cr; 4.6 mL/min for As, Al, and Zn. This configuration effectively reduced polyatomic and doubly charged ion interferences, ensuring high accuracy and precision.

Instrument initiation involved activating the vacuum and water-cooling systems, followed by plasma ignition and stabilization for at least 30 min. Validated ICP-MS operational parameters were as follows: plasma gas flow 18 L/min, nebulizer 1.18 L/min, auxiliary 1.2 L/min, RF power 1600 W, analog stage voltage − 1950 V, pulse stage voltage 1450 V, deflector voltage − 16 V, and cell entrance/exit voltage − 3 V.

#### Sudan dye analysis via GPC

As described in^23^, Sudan dye standards were subjected to a gel permeation chromatography (GPC) clean-up prior to HPLC analysis. A 400 µL aliquot of a 100 mg/kg standard mixture was evaporated to dryness and reconstituted in 4 mL of GPC mobile phase (ethyl acetate: n-hexane, 1:1, v/v). One milliliter of the reconstituted solution was injected into the GPC system, operated at a flow rate of 2 mL/min. The clean-up procedure lasted 30 min, during which eluate fractions were collected at 3-minute intervals (0–3, 3–6, 6–9, 9–12, 12–15, and 15–18 min). This step efficiently separated the target Sudan dyes from matrix interferences, enabling accurate subsequent identification and quantification by HPLC.

#### HPLC analysis of Sudan dyes

As described in^23^, High-performance liquid chromatography (HPLC) was used for the quantification of Sudan dyes. The injection volume was 25 µL, and separation was achieved using a mobile phase composed of acetonitrile and water (95:5, v/v), optimized for hydrophobic compounds. The flow rate was maintained at 1.0 mL/min. Detection was performed using a diode array detector (DAD), with absorbance monitored at 230 nm for all dyes except Sudan Orange G, which was measured at 380 nm. This setup allowed efficient separation and precise quantification of individual Sudan dyes based on their specific absorption characteristic.

#### Pesticide residue analysis by LC-MS/MS

As described in^32,33^, Pesticide residues were analyzed using liquid chromatography–tandem mass spectrometry (LC-MS/MS). Separation was performed on a ZORBAX Eclipse XDB-C18 column (4.6 × 150 mm, 5 μm), suitable for non-polar to moderately polar compounds. Electrospray ionization (ESI) in positive mode was employed, assisted by a nitrogen nebulizer, with gas parameters optimized for maximum sensitivity. The source temperature was set at 400 °C, and the ion spray potential was maintained at 5500 V. Declustering potential and collision energy were individually optimized for each pesticide using standard solutions delivered via a syringe pump.

Quantification and confirmation were performed in Multiple Reaction Monitoring (MRM) mode. The precursor ion (Q1) for each pesticide was selected, fragmented using optimized collision energy, and the resulting product ion (Q3) was monitored to ensure sensitive and selective detection. This approach enabled accurate identification and quantification of multiple pesticide residues in complex matrices.

#### GC-MS/MS analysis of pesticide residues

As described in^32,33^, Gas chromatography–tandem mass spectrometry (GC-MS/MS) was employed for the analysis of pesticide residues. The oven temperature program was set as follows: an initial temperature of 70 °C held for 2 min, ramped to 150 °C at 25 °C/min, then to 200 °C at 3 °C/min, and subsequently to 280 °C at 8 °C/min, with a final hold at 280 °C for 10 min. The total run time for each analysis was 42 min.

Pesticide quantification was performed by comparing sample peak areas to those of standard pesticide solutions. Multi-point calibration curves were employed to enhance accuracy and precision, ensuring reliable quantification of target pesticides in complex sample matrices.

### Health risk Estimation

Assessment of health risks from dietary heavy metal exposure involves a comprehensive evaluation encompassing both non-carcinogenic and carcinogenic effects. This approach is essential for estimating the potential adverse health outcomes associated with the consumption of contaminated foods and beverages^10,14^.

#### Estimation of daily and weekly intake

The risk associated with dietary intake of heavy metals from spices was evaluated by comparing measured metal concentrations with the estimated provisional tolerable daily intake (EPTDI) and estimated provisional tolerable weekly intake (EPTWI), taking into account local consumption patterns. These assessments follow toxicological guidelines and intake limits established by the FAO and WHO. The EPTDI for each metal was calculated by multiplying its mean concentration in the spice by the daily consumption rate, which, according to WHO/GEMS/FOOD^37^, is 2 g/day for zone “G06” (GEMS/Foods, 2012). An average body weight of 60 kg was assumed, following recommendations by the Food and Nutrition Board^38,39^. For long-term exposure evaluation, the EPTWI was compared with the acceptable provisional tolerable weekly intake (APTWI) established by JECFA and the Food and Nutrition Board. The EPTDI was calculated using the following equation:

1 $$\:\varvec{E}\varvec{P}\varvec{T}\varvec{D}\varvec{I}=\frac{{\varvec{F}}_{\varvec{C}}\varvec{*}\:{\varvec{M}}_{\varvec{C}}}{{\varvec{B}}_{\varvec{W}}}\varvec{*}\:{10}^{-3}$$

##### EPTDI

Estimated provisional tolerable daily intake (mg/kg.bw/day).

##### F_C_

Food consumption (g/day).

##### M_C_

Metal concentration (mg/kg).

##### B_W_

Average body weight (Kg).

##### 10^− 3^

The unit conversion factor.

#### Hazard quotient

The Target Hazard Quotient (THQ) is a widely used metric in environmental health and toxicology for assessing non-carcinogenic risks associated with contaminant exposure, including dietary intake of heavy metals. In the context of spices, THQ quantifies the potential health risks arising from regular consumption of contaminated products. THQ values were calculated following the guidelines provided in the USEPA Region III Risk-Based Concentration Tables (US Epa, 2010, 2018)^40,41^.The calculation of THQ is based on the equation provided by the US EPA in 2010 and 2018.

2 $$\:\varvec{T}\varvec{H}\varvec{Q}=\frac{\varvec{C}\varvec{M}\varvec{*}\:\varvec{E}\varvec{D}\varvec{*}\varvec{E}\varvec{F}\varvec{*}\varvec{F}\varvec{I}\varvec{R}}{\varvec{R}\varvec{F}\varvec{D}\varvec{*}{\varvec{B}}_{\varvec{W}}\varvec{*}{\varvec{A}\varvec{T}}_{\varvec{n}}}\varvec{*}\:{10}^{-3}$$

##### CM

Metal concentration (mg/kg).

##### ED

the exposure duration (years).

##### EF

the exposure frequency (day/year).

##### FIR

The food ingestion rate(g/person/day).

##### RFD

The reference dose of the metal (mg/kg/day).

##### B_W_

Average body weight (Kg).

##### 10^− 3^

The unit conversion factor.

##### AT_n_

The average exposure time for noncarcinogens (days).

$$\:\varvec{B}\varvec{W}:\varvec{A}\varvec{v}\varvec{e}\varvec{r}\varvec{a}\varvec{g}\varvec{e}\:\varvec{b}\varvec{o}\varvec{d}\varvec{y}\:\varvec{w}\varvec{e}\varvec{i}\varvec{g}\varvec{h}\varvec{t}\:\left(\varvec{g}\right)\:\:\:\:\:\:\:\:\:\:\:\:\:\:\:\:\:\:\:\:\:\:\:{10}^{-3}:\:\varvec{T}\varvec{h}\varvec{e}\:\varvec{u}\varvec{n}\varvec{i}\varvec{t}\:\varvec{c}\varvec{o}\varvec{n}\varvec{v}\varvec{e}\varvec{r}\varvec{s}\varvec{i}\varvec{o}\varvec{n}\:\varvec{f}\varvec{a}\varvec{c}\varvec{t}\varvec{o}\varvec{r}$$

The Target Hazard Quotient (THQ) is calculated as the ratio of the measured concentration to the oral reference dose, adjusted for exposure duration and frequency, the amount of substance ingested, and body weight. This parameter delineates the duration of exposure and the corresponding risk level. The variables utilized in this computation include Exposure Frequency (EF), representing the number of exposure days per year for non-carcinogenic risk (260 days/year); Exposure Duration (ED), indicating the period for non-cancer risk assessment as adopted by the USEPA (30 years); Food Ingestion Rate (FIR), denoting the daily consumption rate for spices (2 mg/day, reflecting the average intake across all spices samples); Concentration of Metals (CM) in spices (mg/kg); Average Body Weight (WB) (60 kg); Average Time of Exposure (AT_n_), calculated over a period of 30 years (365 days/year); and the Reference Dose (RfD) of the metal (mg/kg/day).

#### Hazard index

The Hazard Index (HI) is used to assess the overall non-carcinogenic risk from multiple contaminants, such as different heavy metals, present in a single source (in this case, spices). It is calculated by summing the individual (THQs) for each contaminant (metal) to get a cumulative risk estimate. The HI provides an indication of the total potential non-carcinogenic risk associated with exposure to multiple pollutants. The (HI) is based on the equation provided by the US EPA in 2010 and 2018^40,41^:

3 $$\:\varvec{H}\varvec{I}=\sum\:{\varvec{T}\varvec{H}\varvec{Q}}_{\varvec{c}\varvec{o}\varvec{n}\varvec{t}\varvec{a}\varvec{m}\varvec{i}\varvec{n}\varvec{a}\varvec{n}\varvec{t}}$$

##### THQ _contaminant_

The target hazard quotient of each contaminant.

### Methods performance and quality control

Method validation followed the principles and acceptance criteria described in Eurachem (2014) and Eurachem/CITAC (2012)^42,43^. Validation was performed separately for heavy metals using ICP-MS and Sudan dyes using HPLC. Validation experiments included determination of instrument sensitivity (LOD, LOQ), calibration linearity, trueness (recovery from spiked samples and certified reference material analysis), precision (repeatability and reproducibility), and estimation of expanded measurement uncertainty. Results were considered acceptable when: R² ≥ 0.995 (practical threshold), spike recoveries within 80–120% (matrix dependent), repeatability RSD ≤ 20%, and CRM Z-scores between − 2 and + 2. **See Tables S1**,** S2 and Figure ****S1**** in the supplementary file.**

The analytical procedures for multi-residue determination of pesticides using GC–MS/MS and LC–MS/MS were validated in accordance with the performance criteria outlined in the European Commission SANTE/11,312/2021 document^44^. The validation parameters included sensitivity (LOD and LOQ), linearity, accuracy through recovery and spiking experiments, and precision expressed as repeatability and reproducibility. **See Table S3 and Figure ****S1**** in the supplementary file**.

### Heavy metals

Limits of detection (LOD) and quantification (LOQ) were determined as method detection and practical quantification limits, respectively, following replicate low-level measurements verified by precision at the LOQ level. LODs were expressed in µg/kg and LOQs in mg/kg. Typical values were As (6.0 µg/kg; 0.02 mg/kg), Pb (5.04 µg/kg; 0.02 mg/kg), and Ni (106.8 µg/kg; 0.5 mg/kg). Calibration curves exhibited excellent linearity with coefficients of determination (R²) ≥ 0.99907 across all analytes. Trueness was evaluated through spike recovery and certified reference material (CRM) analysis. Spike recoveries ranged between 91.1% and 110.7%, while CRM analysis confirmed method accuracy: Mn (certified 61.3 mg/kg) was found at 61.6 mg/kg (100.5% recovery; Z-score = 0.06), and Ni (certified 925 µg/kg) at 961 µg/kg (103.9% recovery; Z-score = 0.22). Precision, expressed as relative standard deviation (RSD), demonstrated good repeatability (1.5–6.2%) and reproducibility (2.4–2.9%). The expanded measurement uncertainty (k = 2) was estimated by combining precision, bias, and other relevant contributions, yielding values typically between 20% and 25%.

### Sudan dyes

For Sudan dyes, method sensitivity was established by estimating LODs and LOQs following the same Eurachem principles. LODs ranged from 100 to 300 µg/kg and LOQs from 1 to 2 mg/kg. Linearity of calibration curves was excellent, with R² values ≥ 0.99968 for all dyes. Method accuracy was verified through spiked sample recoveries (88–108%) and CRM analysis, including CRM 20,124 hot pepper sauce (certified 753 mg/kg; found 750 mg/kg; 99.6% recovery; Z-score = − 0.02) and CRM 20,188 hot sauce (certified 230 mg/kg; found 184 mg/kg; 80.0% recovery; Z-score = − 1). Repeatability RSDs were typically between 2% and 5%, while expanded uncertainties (k = 2) ranged from 17% to 24%, depending on the analyte.

### Pesticide residues

The analytical methods based on GC–MS/MS and LC–MS/MS were successfully validated in accordance with SANTE/11,312/2021 and ISO/IEC 17,025 quality requirements. The experimentally determined limits of detection (LOD = 3 µg/kg) and limits of quantification (LOQ = 0.01 mg/kg) confirmed the high sensitivity of the developed procedures and ensured compliance with the SANTE performance criterion (LOQ ≤ 0.01 mg/kg). Calibration using matrix-matched standards demonstrated excellent linearity across the working range of 0.01–0.5 mg/kg, with correlation coefficients (R²) between 0.991 and 0.999, reflecting strong instrument stability and consistent detector response for all pesticide classes.

Accuracy and trueness were verified through spike-recovery experiments, with recoveries ranging from 70% to 119%, fully satisfying the SANTE acceptance range (70–120%). Method precision, evaluated as relative standard deviation (RSD), showed repeatability between 2% and 11% and reproducibility between 5% and 18%, indicating good consistency under both intra- and inter-day conditions. The expanded measurement uncertainty (k = 2) was estimated at approximately 50%, in line with SANTE performance expectations for multi-residue analysis.

Comprehensive QA/QC procedures were implemented to ensure data reliability and traceability. Blind and verified samples were routinely included to assess analytical performance and instrument stability. Spiked control samples were monitored using X-control charts, with all recovery values remaining within the ± 3σ confidence limits, confirming ongoing analytical precision. Participation in proficiency testing (PT) schemes further validated laboratory competency, yielding Z-scores between − 1.2 and + 0.8 (|z| ≤ 2), which demonstrates satisfactory external performance.

Matrix-matched calibration standards were prepared for each sample type to minimize potential errors arising from matrix-induced signal enhancement or suppression during GC–MS/MS and LC–MS/MS analyses. Matrix effect compensation was applied following established procedures to correct for signal suppression in LC–MS/MS^45^and enhancement in GC–MS/MS^46^. The evaluation involved comparing the response of matrix-matched calibration points (0.05 µg/mL) with solvent-based standards. Differences within ± 20% were considered negligible, while greater deviations were corrected in accordance with the SANTE/11,312/2021 guidelines. To verify the uniformity of matrix effects across the calibration range, three concentration levels (0.01, 0.05, and 0.1 µg/mL) were statistically evaluated using a t-test, which revealed no significant variation among them^47^.

### Statistical analysis

All statistical calculations were performed using Microsoft Excel (Microsoft Office 365). Descriptive statistics, including minimum, maximum, mean, and median concentrations, were calculated for each analyzed element in all analyzed samples. The frequency of detection was determined by categorizing results into three groups: (1) free samples (no detectable concentration), (2) samples below the limit of quantification (LOQ), and (3) samples above the LOQ. Compliance with the maximum permissible limits (MPLs; mg/kg) set by the Egyptian and European standards for spices was also evaluated, and the percentage of samples exceeding these limits was recorded as violated samples.

For health risk assessment, the estimated provisional tolerable daily intake (EPTDI) of each element was calculated based on the average daily consumption of the specific spice and the corresponding mean element concentration. The Target Hazard Quotient (THQ) was determined by dividing the estimated daily intake (EDI) by the oral reference dose (RfD) for each element to evaluate non-carcinogenic health risks. The Hazard Index (HI), representing the combined potential risk from exposure to multiple elements, was calculated as the sum of individual THQ values for all analyzed elements in each spice type. A HI value greater than 1.0 was considered indicative of potential health concern. All computations and data summaries were performed using built-in Excel statistical functions and verified manually for accuracy.

## Results and discussion

### Occurrence of Sudan dyes in spices samples

The screening of 80 spice samples (hot chili, paprika, curry, and cumin) for Sudan dyes revealed varying levels of adulteration. Sudan I was the most frequently detected dye, particularly in hot chili and curry samples, whereas other dyes such as Sudan II, III, and IV appeared at lower frequencies and concentrations. Orange G, Sudan 7b, and Para-Red were not detected in most samples, indicating limited or no use of these illegal colorants within the analyzed batches.

Sudan I showed quantifiable concentrations in all tested spice types. The highest levels were observed in curry (up to 52 mg/kg; mean 8.9 mg/kg) and cumin (up to 14 mg/kg; mean 1.52 mg/kg), whereas paprika and hot chili contained considerably lower concentrations (< 1 mg/kg). All analyzed samples of hot chili and curry (100%) were above the limit of quantification, suggesting the intentional addition of this dye to enhance color intensity. Sudan II contamination was also evident but at generally lower levels. Concentrations ranged from < 0.1 to 14 mg/kg, with mean values between 0.1 and 6.4 mg/kg. The frequency of quantifiable samples was highest in curry (30%) and cumin (25%), confirming a sporadic but non-negligible occurrence. Sudan III and IV were less prevalent. For Sudan III, the majority of samples (≥ 80%) were below the LOQ, with a few hot chili and paprika samples showing trace concentrations up to 0.9 mg/kg. Sudan IV was detected in several paprika, curry, and cumin samples, with maximum values reaching 13 mg/kg (mean = 6.5 mg/kg). The relatively higher detection frequency of Sudan IV in paprika (60% of samples) may reflect its stronger color stability and higher solubility in oil matrices, making it a preferred adulterant where Sudan I is more easily identified. Orange G, Sudan 7b, and Para-Red were not detected (ND) in the analyzed samples. Their absence may indicate a regional preference for specific dye types or an effective restriction on their import and use **(refer** Table 1).

**Table 1:** Occurrence of Sudan dyes in spices samples.

Compound		Sudan dyes concentrations (mg/kg)								Frequency				Free samples				Samples Less than LOQ					Samples Above LOQ					MPL (mg/kg)			The violated elements					The violated samples				
		Minimum		Maximum		Mean		Median		No		%		No		%		No			%		No			%					No			%		No			%	
**SD Ι**	Hot chili	< 0.1		0.9		0.6		0.4		20		100		0		0		0			0		20			100		ND			20			100		80			100	
	Paprika	< 0.1		0.6		0.15		0.1		20		100		0		0		17			85		3			15		ND			20			100						
	Curry	< 0.1		52		8.9		5		20		100		0		0		6			30		14			70		ND			20			100						
	Cumin	< 0.1		14		1.52		0.3		20		100		0		0		5			25		15			75		ND			20			100						
**SD Π**	Hot chili	< 0.1		13		2.68		0.1		5		25		15		75		4			20		1			5		ND			5			25						
	Paprika	< 0.1		12		2.95		0.1		8		40		12		60		2			10		6			30		ND			8			40						
	Curry	< 0.1		14		6.4		5		8		40		12		60		2			10		6			30		ND			8			40						
	Cumin	< 0.1		< 0.1		0.1		0.1		7		35		13		65		7			35		0			0		ND			7			35						
**SD Ш**	Hot chili	< 0.1		0.9		0.37		0.1		4		20		16		80		3			15		1			5		ND			4			20						
	Paprika	< 0.1		0.8		0.5		0.6		3		15		17		85		2			10		1			5		ND			3			15						
	Curry	< 0.1		0.2		0.2		0.2		1		5		19		95		1			5		19			95		ND			1			5						
	Cumin	ND		ND		ND		ND		0		0		0		0		0			0					0		ND			0			0						
**SD ІV**	Paprika	< 0.1		13		6.5		5		20		100		0		0		8			40		12			60		ND			20			100						
	Curry	< 0.1		11		0.9		1.6		20		100		0		0		6			30		14			70		ND			20			100						
	Cumin	< 0.1		11		1.3		0.3		20		100		0		0		4			20		16			80		ND			20			100						
	Paprika	< 0.1		13		1.34		0.1		20		100		0		0		18			90		2			10		ND			20			100						
**Orange G**	Paprika	< 0.1		30		9.5		5		5		25		15		75		1			5		4			20		ND			5			25						
	Curry	< 0.1		23		8.9		8		7		35		13		65		3			15		4			20		ND			7			35						
	Cumin	ND		ND		ND		ND		0		0		20		100		0			0		0			0		ND			0			0						
	Paprika	< 0.1		ND		ND		ND		0		0		0		0		0			0		0			0		ND			0			0						
**Sudan 7b**	Paprika	ND		ND		ND		ND		0		0		20		100		0			0					0		ND			0			0						
	Curry	ND		ND		ND		ND		0		0		20		100		0			0		0			0		ND			0			0						
	Cumin	ND		ND		ND		ND		0		0		20		100		0			0		0			0		ND			0			0						
	Paprika	ND		ND		ND		ND		0		0		0		0		0			0		0			0		ND			0			0						
**Para-Red**	Paprika	ND		ND		ND		ND		0		0		20		100		0			0		0			0		ND			0			0						
	Curry	ND		ND		ND		ND		0		0		20		100		0			0		0			0		ND			0			0						
	Cumin	ND		ND		ND		ND		0		0		20		100		0			0		0			0		ND			0			0						
	Paprika	ND		ND		ND		ND		0		0		0		0		0			0		0			0		ND			0			0						

ND: Not Detected.

Application of the validated analytical method to 21 spice and chili samples revealed widespread contamination with synthetic colorants. Sudan dyes were detected in 17 samples at concentrations ranging from 3.3 to 8709.3 mg/kg, indicating variable but notable adulteration levels across the analyzed products. Para Red was also found in six samples at concentrations between 3.7 and 6.0 mg/kg. The established quantification limits, 0.9–4.8 mg/kg for Sudan dyes and 12.2–19.8 mg/kg for Para Red, confirmed the method’s suitability for trace-level detection^48,49^.

## Occurrence of pesticide residues in spices samples

A total of 461 pesticides were analyzed in the samples. For comparison, the Maximum Residue Limits (MRLs) established by the Codex Alimentarius were applied whenever available. In cases where Codex MRLs were not specified, the European Union MRLs were used as reference standards.

### Pesticide residues in Paprika

Twenty paprika samples were collected from local markets in Egypt. The pesticide residue levels and the percentage of samples exceeding Maximum Residue Limits (MRLs) are summarized in Table 2. All 20 samples (100%) contained detectable pesticide residues, with 9 samples (45%) exceeding the MRLs. The pesticides that exceeded the MRLs were Chlofenapyr, Chlorpyrifos, and Diphenylamine. The most frequently detected pesticides were Chlorfenapyr, followed by Lambda-Cyhalothrin and Difenoconazole.

**Table 2:** Occurrence of pesticide residues in Paprika samples.

Total analysed samples	Number of Contaminated samples	Contamination %	The detected pesticide	frequency	Mean mg/kg	Minimum mg/kg	Maximum mg/kg	MRL mg/kg	Total violated samples	Total violated samples %
**20**	20	100%	Boscalid	3	0.01	0.010	0.01	0.9	9	45%
			Chlofenapyr	18	0.03	< LOQ	0.09	0.05		
			Chlopyriphose	10	0.01	< LOQ	0.035	0.01		
			L-Cyhalothrin	16	0.01	< LOQ	0.035	0.3		
			Cypermethrin	8	0.02	< LOQ	0.03	0.1		
			Deltamethrin	2	0.02	0.02	0.03	15		
			Diazinon	1	0.01	0.01	0.01	0.1		
			Difenconazole	16	0.01	< LOQ	0.02	0.3		
			Diphenylamine	3	0.03	< LOQ	0.07	0.05		
			Malathion	1	0.01	0.01	0.01	0.02		
			Myclobutanile	1	0.01	< LOQ	0.01	0.05		
			Pendimethalin	8	0.01	< LOQ	0.01	0.05		
			Pipronyl-Butoxide	9	0.09	< LOQ	0.478	20		
			Tebuconazole	5	0.01	< LOQ	0.01	0.05		
			Pirimiphos-Me	2	0.13	0.02	0.25	0.5		

LOQ: Limit of Quantification.

### Pesticide residues in curry

Twenty curry samples were collected from the same markets. Table 3 presents the pesticide residue levels and the percentage of samples with residues exceeding the Maximum Residue Limits (MRLs). All 20 samples (100%) contained detectable pesticide residues, with 18 samples (90%) exceeding the MRLs. The compounds found to exceed MRLs included Bifenthrin, Chlorothalonil, Chlorpyrifos, Cyfluthrin, Cypermethrin, Diazinon, Diphenylamine, Fenbuconazole, Fenpropathrin, Kresoxim-Methyl, Malathion, Metalaxyl, Myclobutanil, Penconazole, Pendimethalin, Profenofos, and Propiconazole. The most frequently detected pesticides were Lambda-Cyhalothrin, followed by Cyfluthrin, Kresoxim-Methyl, Chlorothalonil, and Myclobutanil.

**Table 3:** Occurrence of pesticide residues in curry samples.

Total analysed samples	Number of Contaminated samples	Contamination %	The detected pesticide	frequency	Mean mg/kg	Minimum mg/kg	Maximum mg/kg	MRL mg/kg	Total violated samples	Total violated samples %
**20**	20	100%	Bifenithrin	7	0.05	< LOQ	0.15	0.03	18	90%
			Boscalid	8	0.03	< LOQ	0.16	0.9		
			Chlofenapyr	6	0.02	< LOQ	0.04	0.05		
			Chlothalonile	16	0.21	0.019	0.8	0.05		
			Chlopyriphose	5	0.05	< LOQ	0.20	0.01		
			Cyfluthrine	18	0.03	< LOQ	0.16	0.1		
			L-Cyhalothrin	19	0.04	< LOQ	0.16	0.3		
			Cypermethrin	9	0.04	0.014	0.17	0.1		
			Cyproconazole	2	0.01	< LOQ	0.01	0.05		
			Deltamethrin	17	0.04	< LOQ	0.2	15		
			Diazinon	14	0.07	< LOQ	0.18	0.1		
			Difenconazole	5	0.04	< LOQ	0.12	0.3		
			Diniconazole	5	0.03	< LOQ	0.05	0.05		
			Diphenylamine	2	0.05	0.02	0.08	0.05		
			Epoxiconazole	4	0.02	< LOQ	0.06	0.1		
			Ethion	4	0.03	0.010	0.08	5		
			Fenbuconazole	11	0.02	0.011	0.07	0.05		
**20**	20	100%	Fenpropathrin	12	0.04	0.01	0.23	0.02	18	90%
			Flusilazole	1	0.02	0.02	0.02	0.05		
			Hexaconazole	6	0.02	< LOQ	0.05	0.05		
			Isoprothiolane	1	0.01	0.01	0.01	0.01		
			Kresoxim-Me	18	0.06	0.01	0.28	0.05		
			Malathion	14	0.5	< LOQ	0.23	0.02		
			Metalaxyl	13	0.05	< LOQ	0.20	0.05		
			Myclobutanile	16	0.06	0.01	0.32	0.05		
			Oxyflourfen	2	0.01	< LOQ	0.01	0.05		
			Penconazole	4	0.07	< LOQ	0.19	0.05		
			Pendimethalin	8	0.03	< LOQ	0.09	0.05		
			Pipronyl-Butoxide	4	0.04	< LOQ	0.12	20		
			Profenofos	4	0.109	0.026	0.3	0.07		
			Propiconazole	3	0.05	< LOQ	0.13	0.05		
			Tebuconazole	4	0.03	< LOQ	0.05	0.05		
			Pirimiphos-Me	1	0.07	0.07	0.07	0.5		

LOQ: Limit of Quantification.

### Pesticide residues in hot Chili

Twenty hot chili samples were collected from local markets in Egypt. Table 4 outlines the pesticide residue levels and the percentage of samples exceeding Maximum Residue Limits (MRLs). All 20 samples (100%) contained detectable pesticide residues, with 14 samples (70%) exceeding the MRLs. The pesticides that exceeded MRLs included Chlorpropham, Diphenylamine, Kresoxim-Methyl, Penconazole, Propiconazole, Pyridaben, and Bromuconazole. The most frequently detected pesticides were Chlorfenpyr, Chlorpyrifos, Lambda-Cyhalothrin, Cypermethrin, and Propiconazole, followed by Metalaxyl, Difenoconazole, and Penconazole.

**Table 4:** Occurrence of pesticide residues in hot Chili samples.

Total analysed samples	Number of Contaminated samples	Contamination %	The detected pesticide	frequency	Mean mg/kg	Minimum mg/kg	Maximum mg/kg	MRL mg/kg	Total violated samples	Total violated samples %
**20**	20	100%	Boscalid	12	0.05	0.01	0.26	10	14	70%
			Chlorfenpyr	20	0.11	0.02	0.29	3		
			Chlorothalonil	3	0.01	< LOQ	0.01	70		
			Chloroprofam	3	0.04	< LOQ	0.09	0.05		
			Chloropyriphos	20	0.16	0.02	1.46	20		
			Cyfluthrin	10	0.02	< LOQ	0.05	1		
			L. Cyhalothrin	20	0.28	0.02	0.86	3		
			Cypermethrin	20	0.14	0.02	0.53	10		
			Deltamethrin	6	0.03	< LOQ	0.13	15		
			Dicofol	1	0.02	0.02	0.02	0.1		
			Difenconazole	18	0.06	< LOQ	0.22	5		
			Diphenylamine	12	0.03	< LOQ	0.14	0.05		
			Ethion	1	0.06	0.06	0.06	5		
			Fenbuconazole	6	0.01	< LOQ	0.03	2		
			Kresoxim-Me	6	0.05	0.01	0.09	0.05		
			Malathion	3	0.01	< LOQ	0.03	1		
			Metalaxyl	19	0.01	< LOQ	0.04	10		
			Penconazole	18	0.02	< LOQ	0.11	0.05		
**20**	20	100%	Profenofos	17	0.03	< LOQ	0.18	20	14	70%
			Pendimethalin	9	0.01	< LOQ	0.02	0.05		
			Pipronyl-butoxide	17	0.08	< LOQ	0.64	20		
			Propiconazole	20	0.09	< LOQ	0.38	0.05		
			Pyridaben	3	0.06	< LOQ	0.16	0.05		
			Tebuconazole	6	0.06	< LOQ	0.26	10		
			Bromuconazole	1	0.08	0.08	0.08	0.05		
			Tetraconazole	2	0.01	< LOQ	0.01	0.02		

LOQ: Limit of Quantification.

### Pesticide residues in Cumin

Twenty cumin samples were collected from local markets. Table 5 provides the pesticide residue levels and the percentage of samples exceeding Maximum Residue Limits (MRLs). All 20 samples (100%) contained detectable pesticide residues, and all 20 samples (100%) exceeded the MRLs. The pesticides that violated MRLs included Bifenthrin, Boscalid, Chlorfenapyr, Chlorothalonil, Chlorpyrifos, Cyfluthrin, Lambda-Cyhalothrin, Cypermethrin, Cyproconazole, Deltamethrin, Difenoconazole, Diniconazole, Diphenylamine, Fenpropathrin, Hexaconazole, Isoprothiolane, Kresoxim-Methyl, Malathion, Metalaxyl, Myclobutanil, Penconazole, Pendimethalin, Profenofos, Propiconazole, Pyrimethanil, Pyriproxyfen, Tebuconazole, Tetraconazole, Triadimenol, Trifloxystrobin, Iprodione, and Benalaxyl.

**Table 5:** Occurrence of pesticide residues in Cumin samples.

Total analyzed samples	Number of Contaminated samples	Contamination %	The detected pesticide	frequency	Mean mg/kg	Minimum mg/kg	Maximum mg/kg	MRL mg/kg	Total violated samples	Total violated samples %
**20**	20	100%	Bifenithrin	12	0.02	< LOQ	0.06	0.05	20	100%
			Boscalid	19	0.54	< LOQ	2.39	0.9		
			Chlofenapyr	7	0.03	< LOQ	0.06	0.05		
			Chlothalonile	16	0.16	< LOQ	1.50	0.05		
			Chlopyriphose	20	1.15	0.028	3.41	0.01		
			Cyfluthrine	14	0.27	0.03	1.29	0.1		
			L-Cyhalothrin	19	0.50	0.011	2.07	0.3		
			Cypermethrin	20	0.64	< LOQ	2.41	0.1		
			Cyproconazole	9	0.02	< LOQ	0.07	0.05		
			Deltamethrin	12	0.03	< LOQ	0.13	0.1		
			Diazinon	11	0.11	0.01	0.59	5		
			Difenconazole	20	0.80	0.01	2.79	0.3		
			Diniconazole	9	0.06	< LOQ	0.21	0.05		
			Diphenylamine	4	0.0	< LOQ	0.05	0.05		
			Epoxiconazole	11	0.02	< LOQ	0.05	0.1		
			Ethion	15	0.11	< LOQ	0.36	3		
			Fenbuconazole	7	0.01	< LOQ	0.04	0.05		
**20**	20	100%	Fenpropathrin	14	1.17	0.14	14.0	0.02	20	100%
			Flusilazole	1	0.03	0.03	0.03	0.05		
			Hexaconazole	18	0.15	< LOQ	0.67	0.05		
			Isoprothiolane	1	0.02	0.02	0.02	0.01		
			Kresoxim-Me	18	0.25	< LOQ	0.85	0.05		
			Malathion	19	0.23	< LOQ	1.08	0.02		
			Metalaxyl	20	1.19	0.02	5.05	0.05		
			Myclobutanile	16	0.22	< LOQ	0.72	0.05		
			Oxyflourfen	8	0.01	< LOQ	0.02	0.05		
			Penconazole	16	0.12	< LOQ	1.00	0.05		
			Pendimethalin	15	0.02	< LOQ	0.07	0.05		
			Pipronyl-Butoxide	17	0.05	< LOQ	0.18	20		
			Profenofos	19	1.54	0.07	5.95	5		
			Propiconazole	20	0.56	< LOQ	2.54	0.05		
			Pyrimethnil	4	0.04	< LOQ	0.10	0.05		
			Pyriproxyfen	4	0.03	0.01	0.05	0.05		
			Tebuconazole	20	1.20	< LOQ	3.02	1.5		
**20**	20	100%	Tetraconazole	8	0.04	< LOQ	0.12	0.02	20	100%
			Triadiminole	1	0.23	0.23	0.23	0.05		
			Trifloxystrobine	17	0.66	0.02	2.78	0.05		
			Triflumizole	1	0.22	0.22	0.22	0.1		
			Fludioxonil	2	0.02	< LOQ	0.04	4		
			Iprodione	5	0.04	< LOQ	0.18	0.05		
			Pirimiphos-Me	1	0.01	< LOQ	0.01	3		
			Benalaxyl	5	0.13	< LOQ	0.35	0.05		
			Triadimefon	1	0.04	0.04	0.04	0.05		

LOQ: Limit of Quantification.

The most frequently detected pesticides were Chlorpyrifos, Cypermethrin, Difenoconazole, Metalaxyl, and Propiconazole, followed by Boscalid, Lambda-Cyhalothrin, Malathion, and Profenofos.

### Occurrence of various potentially harmful elements in spices samples

The analysis of 80 spice samples (20 each of paprika, hot chili, curry, and cumin) revealed the presence of 13 trace elements. Among the **essential microelements** (Fe, Zn, Cu, Mn, Co, and Ni), all were consistently detected in every analyzed sample (100%). Regarding the **potentially toxic elements** (Pb, Cd, As, Sb, Cr, Hg, and Sn), all were found in all samples except arsenic (As), which was present in 75% (60 samples). Mercury (Hg) and tin (Sn) concentrations were below the limit of quantification (LOQ) in all samples analyzed. All the spice samples tested showed detectable levels of lead (Pb), with 3.75% exceeding the maximum permissible limits (MPLs) set by both Egyptian and European spice standards. The samples that surpassed these limits included hot chili (1.56 mg/kg), curry (1.23 mg/kg), and cumin (0.934 mg/kg). The detailed data for each spice is provided **in the Supplementary Tables S4–S7**, whereas Table 6 summarizes all the findings collectively.

**Table 6:** Occurrence of potentially harmful elements in Cumin samples.

Elements	Paprika			Hot chili			Curry			Cumin			
	**Minimum** **mg/kg**	**Maximum** **mg/kg**	**The violated samples (%)**	**Minimum** **mg/kg**	**Maximum** **mg/kg**	**The violated samples (%)**	**Minimum** **mg/kg**	**Maximum** **mg/kg**	**The violated samples (%)**	**Minimum** **mg/kg**		**Maximum** **mg/kg**	**The violated samples (%)**
As	0.04	0.13	0	< 0.02	0.04	5	< 0.02	< 0.02	5	0.01		0.11	5
Cd	0.07	0.3		0.09	0.23		0.08	0.19		0.14		0.28	
Co	< 0.5	< 0.5		< 0.5	0.96		< 0.5	< 0.5		< 0.5		0.94	
Cr	0.96	3.17		< 0.02	3.08		0.51	3.55		1.15		8.59	
Cu	4.93	9.82		7.25	24.3		4.57	7.85		7.11		22.9	
Fe	406	662		188	1901		146	904		177		1564	
Hg	< 0.05	< 0.05		< 0.05	< 0.05		< 0.05	< 0.05		< 0.05		< 0.05	
Mn	15.5	23.3		14.5	49.7		50.6	166		15		139	
Ni	0.5	1.4		< 0.5	9.71		0.6	3		1.09		4.29	
Pb	0.18	0.75		0.04	1.56		0.13	1.23		0.08		0.93	
Sb	< 0.02	0.03		< 0.02	0.05		< 0.02	< 0.02		< 0.02		< 0.02	
Sn	< 0.5	< 0.5		< 0.5	< 0.5		< 0.5	< 0.5		< 0.5		< 0.5	
Zn	5.46	13.1		7.97	13.8		6.87	15.3		16.2		22.9	

**Paprika samples**:

The analysis of paprika samples showed that Co, Hg, and Sn were below the limit of quantification (LOQ) in all samples. Among the essential elements, Fe, Mn, Cu, Zn, Ni, and Cr were detected in 100% of the samples, with mean concentrations (mg/kg) of 521, 18.3, 7.01, 13.1, 0.89, and 2.06, respectively. For the potentially toxic elements, Cd, Pb, As, and Sb were also present, with mean concentrations of 0.14, 0.29, 0.08, and 0.02 mg/kg, respectively. As was detected in 90% of the samples. All paprika samples contained measurable levels of Pb above the quantification limit; however, none exceeded the maximum permissible limits (MPLs) established by Egyptian and European spice standards (**Table S4 in the Supplementary file**).

**Hot chili samples**:

The analysis of hot chili samples showed that Hg and Sn were below the limit of quantification (LOQ) in all samples. Among the essential microelements, Fe, Mn, Cu, Zn, Co, and Ni were detected in 100% of the samples, with mean concentrations (mg/kg) of 1062, 27.0, 11.3, 10.4, 0.58, and 2.47, respectively. For the potentially toxic elements, Cd, Cr, Pb, and Sb were present in all analyzed samples, with mean concentrations of 0.14, 1.8, 0.52, and 0.02 mg/kg, respectively. As was detected in 80% of the samples, with a mean concentration of 0.02 mg/kg. All hot chili samples contained measurable levels of Pb, and 5% of them (1.56 mg/kg) exceeded the maximum permissible limits (MPLs) established by Egyptian and European spice standards (**Table S5 in the Supplementary file**).

**Curry samples**:

The analysis of curry samples showed that As, Co, Hg, Sb, and Sn were below the limit of quantification (LOQ) in all samples. Among the essential elements, Fe, Mn, Cu, Zn, Ni, and Cr were detected in 100% of the samples, with mean concentrations (mg/kg) of 413, 100.2, 6.49, 11.5, 1.06, and 1.22, respectively. For the potentially toxic elements, Cd and Pb were present in all samples, with mean concentrations of 0.15 and 0.36 mg/kg, respectively. All curry samples contained measurable levels of Pb, and 5% (1.23 mg/kg) of them exceeded the maximum permissible limits (MPLs) established by Egyptian and European spice standards (**Table S6 in the Supplementary file**).

**Cumin samples**:

The analysis of cumin samples showed that Hg, Sb, and Sn were below the limit of quantification (LOQ) in all samples. Among the essential elements, Fe, Mn, Cu, Zn, Co, Ni, and Cr were detected in 100% of the samples, with mean concentrations (mg/kg) of 691, 60.7, 10.8, 20.0, 0.6, 1.78, and 2.8, respectively. For the potentially toxic elements, Cd and Pb were also detected in all samples, with mean concentrations of 0.18 and 0.38 mg/kg, respectively. As was detected in 80% of the samples, with a mean concentration of 0.04 mg/kg. All cumin samples contained measurable levels of Pb, and 5% (0.934 mg/kg) exceeded the maximum permissible limits (MPLs) established by Egyptian and European spice standards (**Table S7 in the Supplementary file**).

### Cross-contamination of spices samples with different contaminants

The results of this study revealed the occurrence of multiple heavy metals, pesticide residues, and banned Sudan dyes in commonly consumed spices collected from Egyptian markets. The presence of these contaminants, even at trace levels, raises important considerations regarding food safety, environmental contamination, and consumer health.

### Heavy metals in spices and comparison with literature

The detected levels of Pb, Cd, and Cu in the analyzed spices were generally comparable to or lower than those reported in previous international studies. Farkas and Mohácsi-Farkas (2010) highlighted the tendency of plant-based spices to accumulate Pb and Cd from the surrounding soil, irrigation water, and atmospheric deposition^50^. The elevated concentrations of Pb (6.24 mg/kg) and Cd (0.20 mg/kg) found in cinnamon in their study underline the natural bioaccumulation potential of spices. The mean Pb concentrations detected in the current study (ranging from 0.29 to 0.52 mg/kg) were below the maximum permissible limits (MPLs) set by Egyptian and European regulations, suggesting that Pb contamination in the analyzed samples remains within acceptable safety thresholds. However, the consistent detection of Pb in all samples (100%) underscores its environmental ubiquity and persistence in the agricultural chain.

The Cu concentrations observed in this study (7.01–11.3 mg/kg) aligned with those reported for black cumin (14–21.3 mg/kg), coriander (20.12–30.3 mg/kg), and fenugreek (11.1–19.9 mg/kg) in Ethiopia and Iraq^51,52^. Conversely, lower Cu concentrations were recorded in turmeric (4.7 mg/kg), coriander (3.55 mg/kg), and white cumin (9.3 mg/kg) from Bangladesh^53^. These variations may be attributed to differences in soil composition, agricultural practices, fertilizer use, and environmental pollution levels across regions. Copper, an essential trace element, plays a critical role in enzymatic activities, yet excessive exposure can lead to oxidative stress and hepatotoxicity^54^.

Iron (Fe) concentrations in the present study (413–1062 mg/kg) were notably lower than those reported by Komy et al. (2005), who found elevated Fe levels in several herbs and spices, including cumin (7450 mg/kg) and black pepper (7950 mg/kg)^55^. The lower Fe levels observed here may reflect differences in soil fertility, irrigation water quality, or the extent of post-harvest contamination. Despite being essential for hemoglobin synthesis, excessive Fe intake may catalyze free radical formation, contributing to oxidative damage in biological systems.

### Sudan dyes in spices and comparison with literature

The detection of Sudan dyes in approximately 60% of analyzed spice samples presents a major public health concern. Although these dyes are banned for food use due to their genotoxic and carcinogenic potential, their illegal application as color enhancers persists in some markets^48^ detected Sudan dye concentrations in chili powder and chili sauce exceeding the European action limit of 500 µg/kg, with values as high as 8709 µg/kg. The concentrations found in this study, while within the same order of magnitude, confirm that the intentional adulteration of spices remains an ongoing issue. Chronic dietary exposure to such azo dyes can lead to oxidative stress, DNA damage, and increased carcinogenic risk, particularly for consumers with high spice consumption habits.

### Pesticide residues in spices and comparison with literature

Our findings are consistent with previous research demonstrating the high prevalence of pesticide residues in spices and herbs. Vázquez et al. (2019) reported that spices such as chili, pepper, turmeric, and cumin frequently contain residues of organophosphates, pyrethroids, and azole fungicides due to their lipophilic nature and large surface area, which enhance the retention of pesticides^56^. Similarly, Natarajan et al. (2022) found that 65–80% of spice samples analyzed in India contained multiple pesticide residues, often exceeding MRLs^57^. Their study emphasized that non-volatile pesticides such as bifenthrin, chlorpyrifos, cypermethrin, and profenofos also detected in our study are common contaminants in dried spices.

The highest contamination rate observed in cumin (100% exceeding MRLs) may be attributed to its cultivation and storage conditions. According to Girame et al. (2021), cumin and coriander are highly susceptible to pesticide accumulation due to their small seed size and oil content, which facilitates the adsorption and retention of lipophilic compounds^58^. Additionally, the absence of stringent residue monitoring programs in spice-producing regions contributes to the persistence of banned or unregulated pesticide use.

The predominance of compounds such as chlorpyrifos, cypermethrin, lambda-cyhalothrin, and difenoconazole in the analyzed samples aligns with their widespread agricultural application in pest control for spice crops. Pyrethroids and organophosphates are particularly common due to their broad-spectrum activity and low cost, although chronic exposure can cause neurotoxic and endocrine-disrupting effects in humans^56^. The detection of difenoconazole, propiconazole, and kresoxim-methyl also reflects the intensive use of azole fungicides for fungal management during spice drying and storage.

### Overall health risk

#### Sudan dyes health risk

Azo dyes are prohibited as food additives due to their potential carcinogenic properties. The International Agency for Research on Cancer (IARC) has classified Sudan I–IV as Group 3carcinogens, meaning they are not classifiable in terms of their carcinogenicity to humans. Similarly, Para Red is considered a potential genotoxic carcinogen^59^.

#### Pesticide’s health risk Estimation

Table 7 presents the estimated average daily intake (EDI) and the hazard index (HI) for pesticide residues detected in spice samples. The average pesticide residue levels were calculated using the monitoring data. The results of the Estimated Average Daily Intake (EADI) calculation are reported separately for each pesticide as part of the exposure assessment. The risk assessment for the daily intake of pesticides, based on pesticide residue data and daily consumption rates for adults, was also calculated.

**Table 7:** Estimated daily intakes of pesticides mg/kg b.w/day).

Detected pesticide	Paprika		Hot chili		Curry		Cumin	
	EDI x10^− 7^	HI	EDI x10^− 7^	HI	EDI x10^− 7^	HI	EDI x10^− 7^	HI
**Bifenithrin**	-	-	-	-	15.3	0.015%	5.96	0.006%
**Boscalid**	3.33	0.001%	16.7	0.004%	10.2	0.003%	181	0.045%
**Chlofenapyr**	9.85	0.003%	37.5	0.013%	5.93	0.002%	8.45	0.003%
**Chlothalonile**	-	-	3.33	0.002%	70.2	0.035%	51.8	0.026%
**Chlopyriphose**	4.75	0.005%	12.3	0.003%	17.2	0.017%	382	0.382%
**Cyfluthrine**	-	-	54.5	0.055%	8.26	0.002%	91.1	0.023%
**L-Cyhalothrin**	4.13	0.002%	8.04	0.002%	11.9	0.006%	167	0.083%
**Cypermethrin**	4.84	0.002%	92.8	0.046%	12.9	0.006%	215	0.107%
**Cyproconazole**	-	-	45.2	0.023%	1.66	0.001%	6.67	0.003%
**Deltamethrin**	6.95	0.007%	9.75	0.010%	12.2	0.012%	8.81	0.009%
**Dicofol**	-	-	7.52	0.038%	22	0.044%	-	-
**Diazinon**	3.62	0.007%	-	-	12.8	0.013%	35.6	0.071%
**Difenconazole**	3.19	0.003%	21.2	0.021%	8.99	0.009%	267	0.267%
**Diniconazole**	-	-	-	-	16.8	0.002%	21.5	0.022%
**Diphenylamine**	9.21	0.001%	9.49	0.001%	7.78	0.008%	7.02	0.001%
**Epoxiconazole**	-	-	-	-	11.1	0.056%	8.10	0.008%
**Ethion**	-	-	19.1	0.096%	7.81	0.003%	35.3	0.176%
**Fenbuconazole**	-	-	4.40E	0.002%	12.8	0.004%	3.85	0.001%
**Fenpropathrin**	-	-	-	-	6.59	0.009%	391	0.130%
**Flusilazole**	-	-	-	-	6.89	0.015%	9.35	0.013%
**Hexaconazole**	-	-	-	-	3.38	0.000%	0.51	0.109%
**Isoprothiolane**	-	-	-	-	19.9	0.001%	6.12	0.001%
**Kresoxim-Me**	-	-	15.9	0.001%	15.7	0.001%	0.83	0.003%
**Malathion**	-	-	4.63	0.000%	15.1	0.002%	77.8	0.003%
**Metalaxyl**	3.33	0.000%	4.01	0.001%	18.9	0.006%	396	0.050%
**Myclobutanile**	1.64	0.001%	-	-	3.27	0.011%	71.7	0.024%
**Oxyflourfen**	-	-	-	-	21.8	0.007%	3.78	0.013%
**Penconazole**	-	-	7.78	0.003%	8.76	0.001%	65.5	0.022%
**Pendimethalin**	3.33	0.000%	3.69	0.000%	12.2	0.001%	5.69	0.001%
**Pipronyl-Butoxide**	30.8	0.002%	0.28	0.001%	36.3	0.012%	17.8	0.001%
**Profenofos**	-	-	9.32	0.003%	15.8	0.002%	512	0.171%
**Propiconazole**	-	-	28.3	0.004%	-	-	186	0.027%
**Pyrimethnil**	-	-	-	-	-	-	12.5	0.001%
**Pyriproxyfen**	-	-	-	-	-	-	10.2	0.001%
**Tebuconazole**	2.44	0.001%	-	-	8.74	0.003%	399	0.133%
**Tetraconazole**	-	-	-	-	-	-	13.9	0.035%
**Triadiminole**	-	-	-	-	-	-	75.6	0.025%
**Trifloxystrobine**	-	-	-	-	-	-	221	0.055%
**Triflumizole**	-	-	-	-	-	-	74.1	0.019%
**Fludioxonil**	-	-	-	-	-	-	8.00	0.000%
**Iprodione**	-	-	-	-	-	-	14.6	0.002%
**Pirimiphos-Me**	43.6	0.015%	-	-	22.6	0.008%	1.94	0.001%
**Benalaxyl**	-	-	-	-	-	-	42.3	0.006%
**Triadimefon**	-	-	-	-	-	-	12.3	0.004%

EDI: Estimated daily intake.

HI: Hazard index.

The highest hazard index (HI) levels for pesticides were found in hot chili samples. The pesticides responsible for the greatest HI values were Ethion (0.096%), Chlorpyrifos (0.055%), Lambda-Cyhalothrin (0.046%), and Dicofol (0.038%). In cumin samples, the most commonly detected pesticides and their associated HI values were Chlorpyrifos (0.382%), Difenconazole (0.267%), Ethion (0.176%), Profenofos (0.171%), Fenpropathrin (0.130%), and Cypermethrin (0.107%).

For paprika samples, the highest HI values were observed for Pirimiphos-Me (0.0145%), followed by Diazinon (0.0072%), Deltamethrin (0.0070%), and Chlorpyrifos (0.0047%). In curry samples, the highest HI values were recorded for Ethion (0.0556%), Diazinon (0.0440%), Chlorothalonil (0.0351%), Chlorpyrifos (0.0172%), Bifenithrin (0.0153%), Difenconazole (0.0128%), and Profenofos (0.0121%) (Table 7).

The present results indicate that long-term exposure of Egyptian consumers to pesticide residues through the consumption of certain raw seasonings does not appear to be associated with significant health risks. However, it is important to note that the current study is limited to a small selection of seasonings. Additionally, the risk assessment for long-term exposure is based on the toxicological evaluation of individual compounds, rather than considering the cumulative exposure to multiple pesticide residues in crops.

Pesticides can have a cumulative “toxic loading” effect over both the short and long term. Each individual accumulates and reacts to chemicals in a way that is biochemically and biographically unique. From birth, people build up a chemical “body burden,” reflecting a combination of childhood and workplace exposures, pesticide residues on food, chemicals in home and personal care products, as well as the quality of air and water in their communities.

The process of dietary pesticide risk assessment involves three major components: estimating pesticide residue levels, estimating food consumption patterns, and characterizing the risk by comparing exposure estimates with toxicological criteria. Each component of this process carries a considerable degree of uncertainty, which may affect the accuracy of the final risk assessment.

#### Potentially toxic elements health risk Estimation

In this study, health risk estimation was conducted by calculating the Estimated Daily and Weekly Intake (EPTDI, EPTWI), the Target Hazard Quotient (THQ), and the Hazard Index (HI). These metrics were used to assess the potential health risks associated with elements in the samples. The EPTDI and EPTWI provide estimates of the average intake of metals on a daily and weekly basis, respectively, while the THQ measures the potential for non-cancer health effects from individual element exposure. The HI, which is the sum of individual THQs for multiple element, was used to assess the overall risk from cumulative potentially toxic element exposure.

#### Estimating the daily and weekly intake

The study selected ten elements As, Cd, Cr, Cu, Fe, Mn, Ni, Pb, Sb, and Zn o estimate daily and weekly intake levels in spice samples. The results indicated that the consumption of these elements did not exceed the Acceptable APTWI for any of the tested spice samples, even at their highest concentrations. The estimated provisional tolerable weekly intake as percentage of accepted provisional tolerable weekly intake for As, Cd, Co, Cr, Cu, Fe, Mn, Ni, Pb, Sb, and Zn varied between 0.03 and 0.20, 0.47–1.01, 0.98–6.91, 0.13–0.48, 1.84–8.45, 0.34–3.03, 0.17–1.89, 0.27–1.46, 0.01–0.03, and 0.05–0.11%, respectively. However, it is not possible to establish acceptable upper intake levels for Cobalt, as current human data does not provide a clear dose-response relationship, according to organizations such as JECFA, the Food and Nutrition Board, EFSA, and the UK Expert Group on Vitamins and Minerals. These findings are summarized in Table 8.

**Table 8:** Estimated weekly intakes of detected elements (mg/kg b.w/day).

Tested elements	APTWI (mg/kg bw/week)	Paprika		Hot chili		Curry		Cumin	
		EPTWI As % of APTWI		EPTWI As % of APTWI		EPTWI As % of APTWI		EPTWI As % of APTWI	
		Mean	Maximum	Mean	Maximum	Mean	Maximum	Mean	Maximum
**As**	1.50 * 10^− 2^	0.13%	0.20%	0.03%	0.06%	-	-	0.07%	0.17%
**Cd**	7.00 * 10^− 3^	0.47%	1.01%	0.46%	0.78%	0.50%	0.63%	0.61%	0.92%
**Cr**	2.90 * 10^− 2^	1.66%	2.55%	1.44%	2.48%	0.98%	2.85%	2.26%	6.91%
**Cu**	1.17	0.14%	0.20%	0.22%	0.48%	0.13%	0.16%	0.22%	0.46%
**Fe**	5.25	2.32%	2.94%	4.72%	8.45%	1.84%	4.02%	3.07%	6.95%
**Mn**	1.28	0.34%	0.43%	0.49%	0.91%	1.83%	3.03%	1.11%	2.53%
**Ni**	1.20 * 10^− 1^	0.17%	0.27%	0.48%	1.89%	0.21%	0.58%	0.35%	0.83%
**Pb**	2.50 * 10^− 2^	0.27%	0.70%	0.49%	1.46%	0.34%	1.15%	0.35%	0.87%
**Sb**	4.20 * 10^− 2^	0.01%	0.02%	0.01%	0.03%	-	-	-	-
**Zn**	4.65	0.05%	0.07%	0.05%	0.07%	0.06%	0.08%	0.10%	0.11%

EPTWI: Estimated provisional tolerable weekly intake.

APTWI: Accepted provisional tolerable weekly intake.

#### Estimating the hazard quotient

Table 9 presents the estimated THQ for individual elements resulting from the consumption of various spices. According to the United States Environmental Protection Agency (USEPA) guidelines, a THQ value of 1 is considered the acceptable limit. The results showed that the THQ values for all elements in the spices were below 1, indicating that there is no significant non-carcinogenic health risk associated with the ingestion of a single heavy metal through the dietary consumption of these spices. This suggests that the consumption of these spices does not pose a health risk concerning the individual elements studied.

**Table 9:** Target hazard quotient (THQ), hazard index (HI) for the intake of analysed elements.

Tested elements	RfDo (mg/kg/day)	Paprika			Hot chili				Curry				Cumin			
		THQ	HI	HI %	THQ		HI	HI %		THQ	HI	HI %		THQ	HI	HI %
**As**	3.00* 10^− 4^	1.04* 10^− 2^	8.38* 10^− 2^	12.39%	2.82* 10^− 3^		2.21* 10^− 1^	1.27%		-	1.08* 10^− 1^	-		8.42* 10^− 3^	2.60* 10^− 1^	3.24%
**Cd**	1.00* 10^− 3^	7.23* 10^− 3^		8.62%	5.52* 10^− 3^			2.50%		4.46* 10^− 3^		4.12%		6.58* 10^− 3^		2.53%
**Co**	3.00* 10^− 4^	-		-	7.60* 10^− 2^			34.38%		-		-		7.44* 10^− 2^		28.61%
**Cr**	3.00* 10^− 3^	2.51* 10^− 2^		29.97%	2.44* 10^− 2^			11.02%		2.81* 10^− 2^		25.96%		6.80* 10^− 2^		26.13%
**Cu**	4.00* 10^− 2^	5.83* 10^− 3^		6.95%	1.44* 10^− 2^			6.51%		4.66* 10^− 3^		4.31%		1.36* 10^− 2^		5.24%
**Fe**	7.00* 10^− 1^	2.25* 10^− 2^		26.81%	6.45* 10^− 2^			29.17%		3.07* 10^− 2^		28.37%		5.31* 10^− 2^		20.40%
**Mn**	1.40* 10^− 1^	3.96* 10^− 3^		4.72%	8.42* 10^− 3^			3.81%		2.82* 10^− 2^		26.07%		2.36* 10^− 2^		9.07%
**Ni**	2.00* 10^− 2^	1.66* 10^− 3^		1.99%	1.15* 10^− 2^			5.22%		3.57* 10^− 3^		3.30%		5.10* 10^− 3^		1.96%
**Pb**	4.00* 10^− 3^	4.48* 10^− 3^		5.34%	9.27* 10^− 3^			4.19%		7.30* 10^− 3^		6.75%		5.55* 10^− 3^		2.13%
**Sb**	4.00* 10^− 4^	1.65* 10^− 3^		1.97%	3.15* 10^− 3^			1.43%		-		-		-		-
**Zn**	3.00* 10^− 1^	1.04* 10^− 3^		1.24%	1.09* 10^− 3^			0.49%		1.21* 10^− 3^		1.12%		1.81* 10^− 3^		0.70%

RfD_0_: Reference dose.

THQ: Target hazard quotient.

HI: Hazard index.

#### Estimating the hazard index

The study’s findings indicate that the cumulative impact of all tested elements, as represented by the Hazard Index (HI), was below the allowable threshold of 1. Co, Cr, Mn, and Fe were identified as the primary contributors to the HI in spices, **as shown in** Table 9. This suggests that there is no significant non-carcinogenic health risk associated with the consumption of these elements through the ingestion of the spices, as all the obtained HI values were below 1. Therefore, the results suggest that the dietary intake of these elements from the tested spices does not pose a health risk.

#### Combined or synergistic risks

Although all calculated THQ and HI values for both metals and pesticides were below 1 (Tables 7, 8 and 9), indicating negligible individual non-carcinogenic risks, these indices assess each contaminant separately and do not account for potential additive or synergistic effects among multiple co-occurring pollutants. The analyzed spice samples, particularly cumin and curry, contained a broad spectrum of contaminants with cumin showing more than 20 pesticide residues (e.g., chlorpyrifos, cypermethrin, profenofos, and difenoconazole) and measurable concentrations of metals such as Fe, Cr, Ni, and Pb. Similarly, curry exhibited diverse pesticide residues along with detectable levels of essential and toxic elements. The co-occurrence of such compounds, even at low individual concentrations, could produce interactive or cumulative effects through shared biochemical or oxidative stress mechanisms. Therefore, while the current results suggest low health concern on a single-compound basis, the potential combined toxicity arising from simultaneous exposure to multiple pesticides and metals cannot be excluded and warrants further evaluation through mixture-based risk models.

#### Cumulative and chronic exposure

The present health risk assessment was conducted using estimated daily (EDI) and weekly (EPTWI) intakes compared to reference values (RfD and APTWI), providing an indication of short-term or independent exposure safety. However, in realistic dietary conditions, consumers are chronically exposed to a variety of spices that may each contain multiple residues of pesticides and trace metals. For instance, cumulative ingestion of Fe, Mn, and Pb from spices such as paprika and hot chili, combined with low-level pesticide residues (e.g., boscalid, chlopyriphose, and tebuconazole), could increase the total exposure burden over time. Although individual HI values for metals (ranging between 0.02% and 29%) and pesticides (typically < 0.5%) were far below 1, chronic exposure through regular consumption may gradually contribute to long-term health effects. This limitation emphasizes the importance of extending the risk assessment framework to include cumulative dietary exposure and chronic intake models, which better reflect real-life consumption patterns and combined contaminant interactions.

## Conclusion

The findings revealed that a considerable proportion of spice samples were contaminated with illegal Sudan dyes, while potentially toxic elements and pesticide residues generally remained within acceptable safety limits. Approximately 60% of the analyzed samples contained five Sudan dyes at concentrations above the quantification limit of 0.1 mg/kg, despite their regulatory ban. The estimated Target Hazard Quotient (THQ) values for all analyzed elements were below 1, indicating no significant non-carcinogenic health risk associated with metal or pesticide exposure. The cumulative exposure assessment indicated that the overall risk was within acceptable limits under the tested conditions. However, given the limited sample size and the absence of long-term cumulative exposure data, these findings should be interpreted with caution. While the detected levels of heavy metals and pesticide residues may not pose an immediate health threat, the recurrent detection of Sudan dyes in commonly consumed spices underscores a persistent safety concern and highlights the urgent need for enhanced regulatory surveillance and stricter enforcement measures. Continuous consumption over time may lead to gradual accumulation and potential long-term health impacts. Therefore, it is essential to expand the current risk assessment framework to incorporate cumulative exposure and chronic intake models that better represent actual dietary habits and multi-contaminant interaction.

## Supplementary Information

Below is the link to the electronic supplementary material.

Supplementary Material 1

Supplementary Material 2

## Data Availability

The datasets used and/or analyzed during the current study available from the corresponding author on reasonable request.
