# Supplementary figures and images for: Health risk assessment of Sudan dyes, toxic elements, and pesticide residues in Egyptian spices
Source: Sci Rep. 2025 Dec 23;15:44332. doi: 10.1038/s41598-025-31386-3 (PMC12727808; doi:10.1038/s41598-025-31386-3)

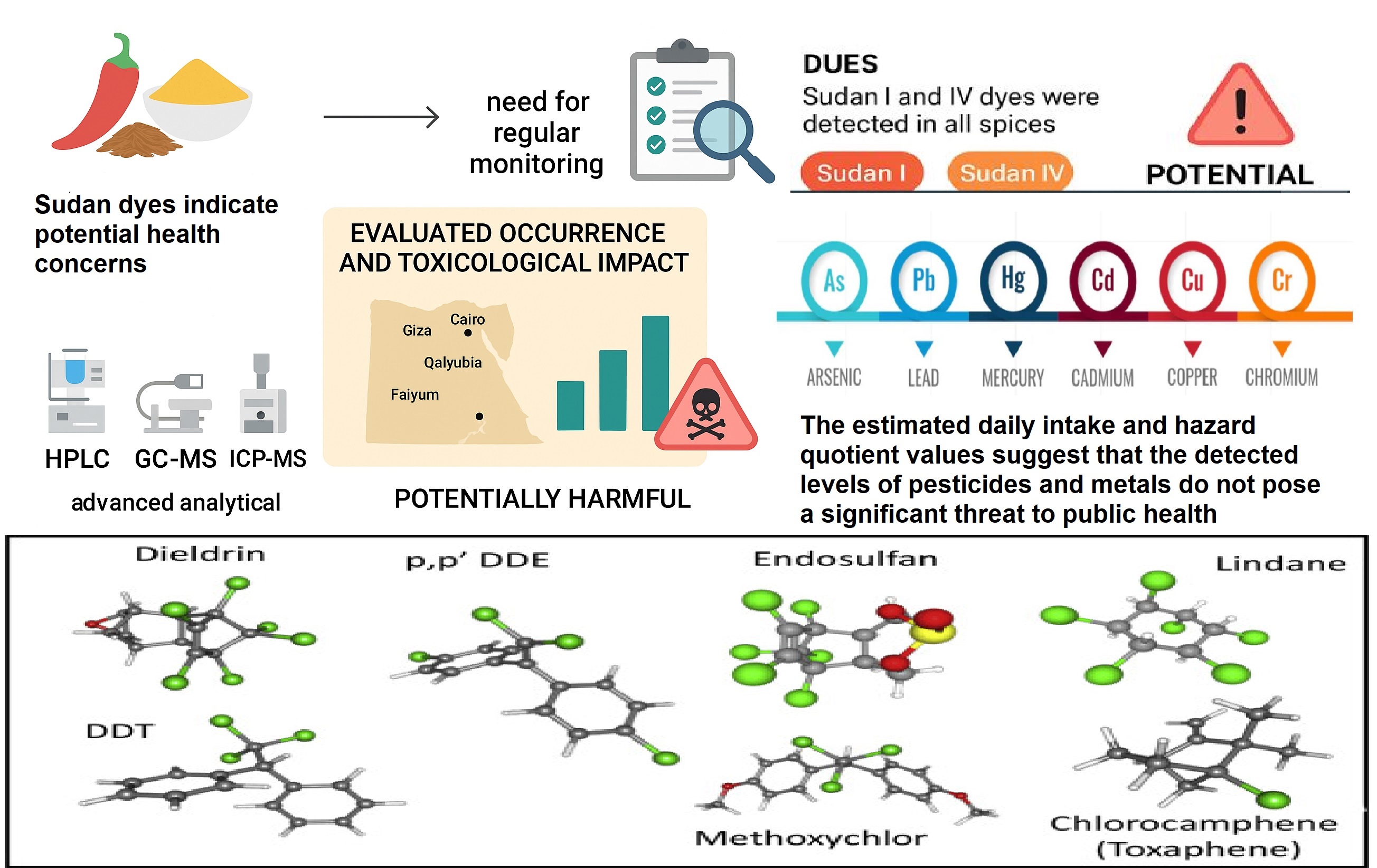

Supplement: Supplementary file 1 — Supplementary Material 1 [file 41598_2025_31386_MOESM1_ESM.jpg]
