# Supplementary material for: Health risk assessment of Sudan dyes, toxic elements, and pesticide residues in Egyptian spices
Source: Sci Rep. 2025 Dec 23;15:44332. doi: 10.1038/s41598-025-31386-3 (PMC12727808; doi:10.1038/s41598-025-31386-3)
Supplement: Supplementary file 2 — Supplementary Material 2 [file 41598_2025_31386_MOESM2_ESM.docx]

**Supplementary Data**

**Health Risk Assessment of Sudan Dyes, Toxic Elements, and Pesticide Residues in Egyptian Spices**

Mahmoud M. Ghuniem^1*^, Hoda M. Refai^1^, Khaled Rabie^1^

^1^Ministry of Agriculture and Land Reclamation, Agricultural Research Center, Central Laboratory of Residue Analysis of Pesticides and Heavy Metals in Foods (QCAP Egypt), 7-Nadi El-said Street, Dokki, Giza, Egypt, P.O. 12311.

* Corresponding author.

**Mahmoud M. Ghuniem:**

E-mail: Mahmoud.ghuniem@qcap-egypt.com & Mahmoud_ghuniem88@yahoo.com

Telephone number: +201008755289

Fax: +202-37611216

ORCID ID: 0000-0001-7071-9190

Table S1. Methods performance for heavy metals and Sudan dayes

| **Elements** | **Estimated**  **LODs (µg/kg)** | **Practical**  **LOQs (mg/kg)** | **Recovery ranges (%)** | **Method**  **Linearity**  **R^2^** | **RSD (%)** | | | **Expanded**  **Uncertainty (%)** | |  |
| --- | --- | --- | --- | --- | --- | --- | --- | --- | --- | --- |
|  |  |  |  |  | | **Repeatability** | **Reproducibility** | |  | |
| **As** | 6.0 | 0.02 | 94.8 – 105.3 | 0.99986 | | 2.6 | 2.9 | | 21.0 | |
| **Cd** | 5.02 | 0.02 | 99.8 – 110.7 | 0.99990 | | 1.5 | 2.4 | | 20.7 | |
| **Co** | 20.5 | 0.5 | 107.9 – 112.2 | 0.99998 | | 2.4 | 3.5 | | 21.4 | |
| **Cr** | 55.9 | 0.5 | 101.9 – 111.0 | 0.99998 | | 2.2 | 2.7 | | 21.0 | |
| **Cu** | 78.5 | 0.5 | 97.5 – 103.5 | 0.99998 | | 3.0 | 4.2 | | 21.7 | |
| **Fe** | 21.3 | 0.5 | 89.9 – 110.0 | 0.99907 | | 6.2 | 7.0 | | 25.1 | |
| **Hg** | 7.03 | 0.05 | 86.4 – 108.1 | 0.99996 | | 3.1 | 4.3 | | 21.6 | |
| **Mn** | 19.3 | 0.5 | 102.3 – 115.1 | 0.99999 | | 2.6 | 3.9 | | 21.8 | |
| **Ni** | 106.8 | 0.5 | 99.3 – 107.6 | 0.99997 | | 2.4 | 6.3 | | 24.5 | |
| **Pb** | 5.04 | 0.02 | 91.1 – 98.6 | 0.99997 | | 1.6 | 2.8 | | 20.5 | |
| **Sb** | 4.45 | 0.02 | 96.7 – 113.6 | 0.99994 | | 3.9 | 7.6 | | 25.8 | |
| **Sn** | 48.8 | 0.5 | 88.2 – 102.1 | 0.99999 | | 1.7 | 3.1 | | 21.4 | |
| **Zn** | 33.4 | 0.5 | 94.0 – 99.6 | 0.99993 | | 1.6 | 2.7 | | 20.9 | |
| **SD Ι** | 100 | 1 | 90.0 – 105.0 | 0.99988 | | 4 | 8 | | 17 | |
| **SD Π** | 100 | 1 | 88.0 – 101.0 | 0.99971 | | 3 | 8 | | 17 | |
| **SD Ш** | 100 | 2 | 92.0 – 108.0 | 0.99976 | | 3 | 9 | | 19 | |
| **SD ІV** | 100 | 1 | 88.0 – 102.0 | 0.99968 | | 5 | 11 | | 24 | |
| **Orange G** | 200 | 2 | 94.0 – 106.0 | 0.99987 | | 2 | 9 | | 18 | |
| **Sudan 7b** | 300 | 2 | 95.0 – 107.0 | 0.99996 | | 2 | 9 | | 18 | |
| **Para-Red** | 200 | 2 | 96.0 – 105.0 | 0.99978 | | 3 | 8 | | 17 | |

**LODs:** Limit of detections

**LOQs:** Limits of quantiﬁcations

**R^2^ :** Correlations coeﬃcients

**RSD:** Relative standard deviation

Table S2: Result of different certified reference materials.

| CRMs | Compound | Certified value | Found value | Unit | Recovery % | Z-score |
| --- | --- | --- | --- | --- | --- | --- |
| 20188 Hot Sauce | **SD Ι** | 230 | 184 | mg/kg | **80.00%** | **-1** |
|  | **SD Ш** | 419 | 363 | mg/kg | **86.60%** | **-1.15** |
| 20124 Hot Pepper Sauce | **SD Π** | 753 | 750 | mg/kg | **99.60%** | **-0.02** |
|  | **Sudan 7b** | 894 | 900 | mg/kg | **100.70%** | **0.04** |
| IPE 907 Spinach powder | **Mn** | 61.3 | 61.6 | mg/kg | **100.5%** | **0.06** |
|  | **Ni** | 925 | 961 | µg/kg | **103.9%** | **0.22** |
|  | **Cu** | 10.2 | 10.2 | mg/kg | **100.0%** | **0.00** |
|  | **Cr** | 2100 | 2140 | µg/kg | **101.9%** | **0.08** |
|  | **Fe** | 506 | 454.7 | mg/kg | **89.9%** | **-0.73** |
|  | **Zn** | 158 | 165.9 | mg/kg | **105.0%** | **0.59** |
|  | **As** | 338 | 328.8 | µg/kg | **97.3%** | **-0.15** |
|  | **Cd** | 1210 | 1150 | µg/kg | **95.0%** | **-0.38** |
|  | **Pb** | 2040 | 2000 | µg/kg | **98.0%** | **-0.11** |
| IPE 200 Maize powder | **Mn** | 15.2 | 16.2 | mg/kg | **106.6%** | **0.11** |
|  | **Cu** | 2.59 | 2.55 | mg/kg | **98.5%** | **-0.04** |
|  | **Fe** | 86 | 84.0 | mg/kg | **97.7%** | **-0.02** |
|  | **Zn** | 15.9 | 15.1 | mg/kg | **95.1%** | **-0.32** |
|  | **Cd** | 38.8 | 39.7 | µg/kg | **102.3%** | **0.17** |
|  | **Pb** | 290 | 315 | µg/kg | **108.6%** | **0.20** |

Table S3. Methods performance for Pesticides

| Pesticides | Method | LOD  µg/kg | LOQ  mg/kg | Recovery ranges  (%) | RSD (%) | | Linearity  R^2^ |
| --- | --- | --- | --- | --- | --- | --- | --- |
|  |  |  |  |  | **Repeatability** | **Reproducibility** |  |
| Bifenithrin | GC-MS/MS | 3 | 0.01 | 76 - 90% | 3% | 8% | 0.996 |
| Boscalid | LC-MS/MS | 3 | 0.01 | 84 - 89% | 5% | 16% | 0.998 |
| Chlofenapyr | GC-MS/MS | 3 | 0.01 | 89 - 104% | 4% | 17% | 0.995 |
| Chlothalonile | GC-MS/MS | 3 | 0.01 | 70 - 88% | 6% | 12% | 0.994 |
| Chlopyriphose | GC-MS/MS | 3 | 0.01 | 90 - 105% | 3% | 10% | 0.999 |
| Cyfluthrine | GC-MS/MS | 3 | 0.01 | 107 - 112% | 4% | 5% | 0.995 |
| L-Cyhalothrin | GC-MS/MS | 3 | 0.01 | 87 - 111% | 5% | 16% | 0.998 |
| Cypermethrin | GC-MS/MS | 3 | 0.01 | 107 - 119% | 4% | 5% | 0.992 |
| Cyproconazole | LC-MS/MS | 3 | 0.01 | 77 - 85% | 4% | 13% | 0.992 |
| Deltamethrin | GC-MS/MS | 3 | 0.01 | 89 - 110% | 8% | 16% | 0.994 |
| Dicofol | GC-MS/MS | 3 | 0.01 | 93 - 109% | 8% | 16% | 0.996 |
| Diazinon | GC-MS/MS | 3 | 0.01 | 97 - 105% | 2% | 8% | 0.999 |
| Difenconazole | LC-MS/MS | 3 | 0.01 | 77 - 84% | 5% | 10% | 0.995 |
| Diniconazole | LC-MS/MS | 3 | 0.01 | 79 - 84% | 4% | 15% | 0.995 |
| Diphenylamine | GC-MS/MS | 3 | 0.01 | 96 - 103% | 3% | 8% | 0.997 |
| Epoxiconazole | LC-MS/MS | 3 | 0.01 | 76 - 84% | 4% | 13% | 0.999 |
| Ethion | LC-MS/MS | 3 | 0.01 | 94 - 100% | 2% | 9% | 0.996 |
| Fenbuconazole | GC-MS/MS | 3 | 0.01 | 70 - 78% | 11% | 13% | 0.993 |
| Fenpropathrin | GC-MS/MS | 3 | 0.01 | 93 - 117% | 4% | 16% | 0.996 |
| Flusilazole | LC-MS/MS | 3 | 0.01 | 76 - 87% | 3% | 13% | 0.998 |
| Hexaconazole | GC-MS/MS | 3 | 0.01 | 82 - 110% | 10% | 18% | 0.997 |
| Isoprothiolane | GC-MS/MS | 3 | 0.01 | 91 - 100% | 2% | 8% | 0.999 |
| Kresoxim-Me | LC-MS/MS | 3 | 0.01 | 88 - 100% | 2% | 7% | 0.998 |
| Malathion | GC-MS/MS | 3 | 0.01 | 101 - 116% | 4% | 16% | 0.999 |
| Metalaxyl | LC-MS/MS | 3 | 0.01 | 89 - 98% | 5% | 14% | 0.994 |
| Myclobutanile | LC-MS/MS | 3 | 0.01 | 76 - 83% | 3% | 15% | 0.997 |
| Oxyflourfen | GC-MS/MS | 3 | 0.01 | 87 - 101% | 3% | 12% | 0.998 |
| Penconazole | LC-MS/MS | 3 | 0.01 | 78 - 89% | 3% | 12% | 0.999 |
| Pendimethalin | GC-MS/MS | 3 | 0.01 | 89 - 102% | 4% | 11% | 0.999 |
| Pipronyl-Butoxide | LC-MS/MS | 3 | 0.01 | 97 - 106% | 3% | 12% | 0.998 |
| Profenofos | GC-MS/MS | 3 | 0.01 | 95 - 104% | 3% | 6% | 0.998 |
| Propiconazole | LC-MS/MS | 3 | 0.01 | 87 - 91% | 5% | 15% | 0.996 |
| Pyrimethnil | LC-MS/MS | 3 | 0.01 | 83 - 92% | 6% | 9% | 0.997 |
| Pyriproxyfen | LC-MS/MS | 3 | 0.01 | 90 - 99% | 4% | 14% | 0.999 |
| Tebuconazole | GC-MS/MS | 3 | 0.01 | 82 - 88% | 5% | 14% | 0.999 |
| Tetraconazole | GC-MS/MS | 3 | 0.01 | 90 - 99% | 5% | 14% | 0.994 |
| Triadiminole | GC-MS/MS | 3 | 0.01 | 83 - 97% | 6% | 15% | 0.992 |
| Trifloxystrobine | LC-MS/MS | 3 | 0.01 | 92 - 102% | 2% | 8% | 0.996 |
| Triflumizole | GC-MS/MS | 3 | 0.01 | 85 - 91% | 3% | 9% | 0.991 |
| Fludioxonil | GC-MS/MS | 3 | 0.01 | 83 - 93% | 3% | 11% | 0.993 |
| Iprodione | GC-MS/MS | 3 | 0.01 | 84 - 106% | 9% | 12% | 0.992 |
| Pirimiphos-Me | LC-MS/MS | 3 | 0.01 | 98 - 107% | 2% | 10% | 0.999 |
| Benalaxyl | LC-MS/MS | 3 | 0.01 | 98 - 108% | 4% | 12% | 0.998 |
| Triadimefon | GC-MS/MS | 3 | 0.01 | 91 - 98% | 3% | 14% | 0.999 |

Table S4: Occurrence of potentially harmful elements in paprika samples

| **Elements** | **Element’s concentrations (mg/kg)** | | | | **Frequency** | | **Free samples** | | **Samples Less than LOQ** | | **Samples Above LOQ** | | **MPL (mg/kg)** | **The violated elements** | |
| --- | --- | --- | --- | --- | --- | --- | --- | --- | --- | --- | --- | --- | --- | --- | --- |
|  | **Minimum** | **Maximum** | **Mean** | **Median** | **No** | **%** | **No** | **%** | **No** | **%** | **No** | **%** |  | **No** | **%** |
| **As** | 0.04 | 0.13 | 0.08 | 0.08 | 18 | 90.0% | 2 | 10.0% | 0 | 0.0% | 18 | 90.0% | - | - | - |
| **Cd** | 0.07 | 0.30 | 0.14 | 0.14 | 20 | 100% | 0 | 0.0% | 0 | 0.0% | 20 | 100% | - | - | - |
| **Co** | < 0.5 | < 0.5 | 0.50 | 0.50 | 20 | 100% | 0 | 0.0% | 20 | 100% | 0 | 0.0% | - | - | - |
| **Cr** | 0.96 | 3.17 | 2.06 | 2.04 | 20 | 100% | 0 | 0.0% | 0 | 0.0% | 20 | 100% | - | - | - |
| **Cu** | 4.93 | 9.82 | 7.01 | 6.95 | 20 | 100% | 0 | 0.0% | 0 | 0.0% | 20 | 100% | - | - | - |
| **Fe** | 406 | 662 | 521 | 516 | 20 | 100% | 0 | 0.0% | 0 | 0.0% | 20 | 100% | - | - | - |
| **Hg** | < 0.05 | < 0.05 | 0.05 | 0.05 | 20 | 100% | 0 | 0.0% | 20 | 100% | 0 | 0.0% | - | - | - |
| **Mn** | 15.5 | 23.3 | 18.8 | 18.4 | 20 | 100.% | 0 | 0.0% | 0 | 0.0% | 20 | 100% | - | - | - |
| **Ni** | 0.50 | 1.40 | 0.89 | 0.85 | 20 | 100% | 0 | 0.0% | 0 | 0.0% | 20 | 100% | - | - | - |
| **Pb** | 0.18 | 0.75 | 0.29 | 0.25 | 20 | 100% | 0 | 0.0% | 0 | 0.0% | 20 | 100% | 0.9 | 0 | 0.0% |
| **Sb** | < 0.02 | 0.03 | 0.02 | 0.02 | 20 | 100% | 0 | 0.0% | 16 | 80.0% | 4 | 20.0% | - | - | - |
| **Sn** | < 0.5 | < 0.5 | 0.50 | 0.50 | 20 | 100% | 0 | 0.0% | 20 | 100% | 0 | 0.0% | - | - | - |
| **Zn** | 5.46 | 13.1 | 9.13 | 9.15 | 20 | 100% | 0 | 0.0% | 0 | 0.0% | 20 | 100% | - | - | - |

**ND: Not detectable**

**MPL: Maximum permissible limits**

Table S5: Occurrence of potentially harmful elements in hot chili samples

| **Elements** | **Element’s concentrations (mg/kg)** | | | | **Frequency** | | **Free samples** | | **Samples Less than LOQ** | | **Samples Above LOQ** | | **MPL (mg/kg)** | **The violated elements** | | **The violated samples** | |
| --- | --- | --- | --- | --- | --- | --- | --- | --- | --- | --- | --- | --- | --- | --- | --- | --- | --- |
|  | **Minimum** | **Maximum** | **Mean** | **Median** | **No** | **%** | **No** | **%** | **No** | **%** | **No** | **%** |  | **No** | **%** | **No** | **%** |
| **As** | < 0.02 | 0.04 | 0.02 | 0.02 | 16 | 80.0% | 4 | 20.0% | 0 | 0.0% | 16 | 80.0% | - | - | - | 1 | 5.0% |
| **Cd** | 0.09 | 0.23 | 0.14 | 0.14 | 20 | 100% | 0 | 0.0% | 0 | 0.0% | 20 | 100% | - | - | - |  |  |
| **Co** | < 0.5 | 0.96 | 0.58 | 0.50 | 20 | 100% | 0 | 0.0% | 10 | 50.0% | 10 | 50.0% | - | - | - |  |  |
| **Cr** | < 0.02 | 3.08 | 1.80 | 1.88 | 20 | 100% | 0 | 0.0% | 1 | 5.0% | 19 | 95.0% | - | - | - |  |  |
| **Cu** | 7.25 | 24.3 | 11.3 | 10.2 | 20 | 100% | 0 | 0.0% | 0 | 0.0% | 20 | 100% | - | - | - |  |  |
| **Fe** | 188 | 1901 | 1062 | 1153 | 20 | 100% | 0 | 0.0% | 0 | 0.0% | 20 | 100% | - | - | - |  |  |
| **Hg** | < 0.05 | < 0.05 | 0.05 | 0.05 | 20 | 100% | 0 | 0.0% | 20 | 100% | 0 | 0.0% | - | - | - |  |  |
| **Mn** | 14.5 | 49.7 | 27.0 | 25.7 | 20 | 100% | 0 | 0.0% | 0 | 0.0% | 20 | 100% | - | - | - |  |  |
| **Ni** | < 0.5 | 9.71 | 2.47 | 1.79 | 20 | 100% | 0 | 0.0% | 1 | 5.0% | 19 | 95.0% | - | - | - |  |  |
| **Pb** | 0.04 | 1.56 | 0.52 | 0.48 | 20 | 100% | 0 | 0.0% | 0 | 0.0% | 20 | 100% | 0.9 | 1 | 5.0% |  |  |
| **Sb** | < 0.02 | 0.05 | 0.02 | 0.02 | 20 | 100% | 0 | 0.0% | 13 | 65.0% | 7 | 35.0% | - | - | - |  |  |
| **Sn** | < 0.5 | < 0.5 | 0.50 | 0.50 | 20 | 100% | 0 | 0.0% | 20 | 100% | 0 | 0.0% | - | - | - |  |  |
| **Zn** | 7.97 | 13.8 | 10.4 | 10.2 | 20 | 100% | 0 | 0.0% | 0 | 0.0% | 20 | 100% | - | - | - |  |  |

**ND: Not detectable**

**MPL: Maximum permissible limits**

Table S6: Occurrence of potentially harmful elements in curry samples

| **Elements** | **Element’s concentrations (mg/kg)** | | | | **Frequency** | | **Free samples** | | **Samples Less than LOQ** | | **Samples Above LOQ** | | **MPL (mg/kg)** | **The violated elements** | | | **The violated samples** | | |
| --- | --- | --- | --- | --- | --- | --- | --- | --- | --- | --- | --- | --- | --- | --- | --- | --- | --- | --- | --- |
|  | **Minimum** | **Maximum** | **Mean** | **Median** | **No** | **%** | **No** | **%** | **No** | **%** | **No** | **%** |  | **No** | **%** | **No** | | **%** |  |
| **As** | < 0.02 | < 0.02 | 0.02 | 0.02 | 8 | 40.0% | 12 | 60.0% | 8 | 40.0% | 0 | 0.0% | - | - | - | 1 | | 5.0% |  |
| **Cd** | 0.08 | 0.19 | 0.15 | 0.15 | 20 | 100% | 0 | 0.0% | 0 | 0.0% | 20 | 100% | - | - | - |  |  |  |  |
| **Co** | < 0.5 | < 0.5 | 0.50 | 0.50 | 20 | 100% | 0 | 0.0% | 20 | 100% | 0 | 0.0% | - | - | - |  |  |  |  |
| **Cr** | 0.51 | 3.55 | 1.22 | 1.04 | 20 | 100% | 0 | 0.0% | 1 | 5.0% | 19 | 95.0% | - | - | - |  |  |  |  |
| **Cu** | 4.57 | 7.85 | 6.49 | 6.77 | 20 | 100% | 0 | 0.0% | 0 | 0.0% | 20 | 100% | - | - | - |  |  |  |  |
| **Fe** | 146 | 904 | 413 | 378 | 20 | 100% | 0 | 0.0% | 0 | 0.0% | 20 | 100% | - | - | - |  |  |  |  |
| **Hg** | < 0.05 | < 0.05 | 0.05 | 0.05 | 20 | 100% | 0 | 0.0% | 20 | 100% | 0 | 0.0% | - | - | - |  |  |  |  |
| **Mn** | 50.6 | 166 | 100 | 97.4 | 20 | 100% | 0 | 0.0% | 0 | 0.0% | 20 | 100% | - | - | - |  |  |  |  |
| **Ni** | 0.60 | 3.00 | 1.06 | 0.92 | 20 | 100% | 0 | 0.0% | 0 | 0.0% | 20 | 100% | - | - | - |  |  |  |  |
| **Pb** | 0.13 | 1.23 | 0.36 | 0.31 | 20 | 100% | 0 | 0.0% | 0 | 0.0% | 20 | 100% | 0.9 | 1 | 5.0% |  |  |  |  |
| **Sb** | < 0.02 | < 0.02 | 0.02 | 0.02 | 20 | 100% | 0 | 0.0% | 20 | 100% | 0 | 0.0% | - | - | - |  |  |  |  |
| **Sn** | < 0.5 | < 0.5 | 0.50 | 0.50 | 20 | 100% | 0 | 0.0% | 20 | 100% | 0 | 0.0% | - | - | - |  |  |  |  |
| **Zn** | 6.87 | 15.3 | 11.5 | 12.0 | 20 | 100% | 0 | 0.0% | 0 | 0.0% | 20 | 100% | - | - | - |  |  |  |  |

**ND: Not detectable**

**MPL: Maximum permissible limits**

Table S7: Occurrence of potentially harmful elements in cumin samples

| **Elements** | **Element’s concentrations (mg/kg)** | | | | **Frequency** | | **Free samples** | | **Samples Less than LOQ** | | **Samples Above LOQ** | | **MPL (mg/kg)** | **The violated elements** | | **The violated samples** | |
| --- | --- | --- | --- | --- | --- | --- | --- | --- | --- | --- | --- | --- | --- | --- | --- | --- | --- |
|  | **Minimum** | **Maximum** | **Mean** | **Median** | **No** | **%** | **No** | **%** | **No** | **%** | **No** | **%** |  | **No** | **%** | **No** | **%** |
| **As** | 0.01 | 0.11 | 0.04 | 0.04 | 18 | 90.0% | 2 | 10.0% | 0 | 0.0% | 18 | 90.0% | - | - | - | 1 | 5.0% |
| **Cd** | 0.14 | 0.28 | 0.18 | 0.18 | 20 | 100% | 0 | 0.0% | 0 | 0.0% | 20 | 100% | - | - | - |  |  |
| **Co** | < 0.5 | 0.94 | 0.60 | 0.50 | 20 | 100% | 0 | 0.0% | 14 | 70.0% | 6 | 30.0% | - | - | - |  |  |
| **Cr** | 1.15 | 8.59 | 2.80 | 2.67 | 20 | 100% | 0 | 0.0% | 0 | 0.0% | 20 | 100% | - | - | - |  |  |
| **Cu** | 7.11 | 22.9 | 10.8 | 10.3 | 20 | 100% | 0 | 0.0% | 0 | 0.0% | 20 | 100% | - | - | - |  |  |
| **Fe** | 177 | 1564 | 692 | 581 | 20 | 100% | 0 | 0.0% | 0 | 0.0% | 20 | 100% | - | - | - |  |  |
| **Hg** | < 0.05 | < 0.05 | 0.05 | 0.05 | 20 | 100% | 0 | 0.0% | 20 | 100% | 0 | 0.0% | - | - | - |  |  |
| **Mn** | 15 | 139 | 60.7 | 59.1 | 20 | 100% | 0 | 0.0% | 0 | 0.0% | 20 | 100% | - | - | - |  |  |
| **Ni** | 1.09 | 4.29 | 1.78 | 1.69 | 20 | 100% | 0 | 0.0% | 0 | 0.0% | 20 | 100% | - | - | - |  |  |
| **Pb** | 0.08 | 0.93 | 0.38 | 0.36 | 20 | 100% | 0 | 0.0% | 0 | 0.0% | 20 | 100% | 0.9 | 1 | 5.0% |  |  |
| **Sb** | < 0.02 | < 0.02 | 0.02 | 0.02 | 20 | 100% | 0 | 0.0% | 20 | 100% | 0 | 0.0% | - | - | - |  |  |
| **Sn** | < 0.5 | < 0.5 | 0.50 | 0.50 | 20 | 100% | 0 | 0.0% | 20 | 100% | 0 | 0.0% | - | - | - |  |  |
| **Zn** | 16.2 | 22.9 | 20 | 19.5 | 20 | 100% | 0 | 0.0% | 0 | 0.0% | 20 | 100% | - | - | - |  |  |

**ND: Not detectable**

**MPL: Maximum permissible limits**

**
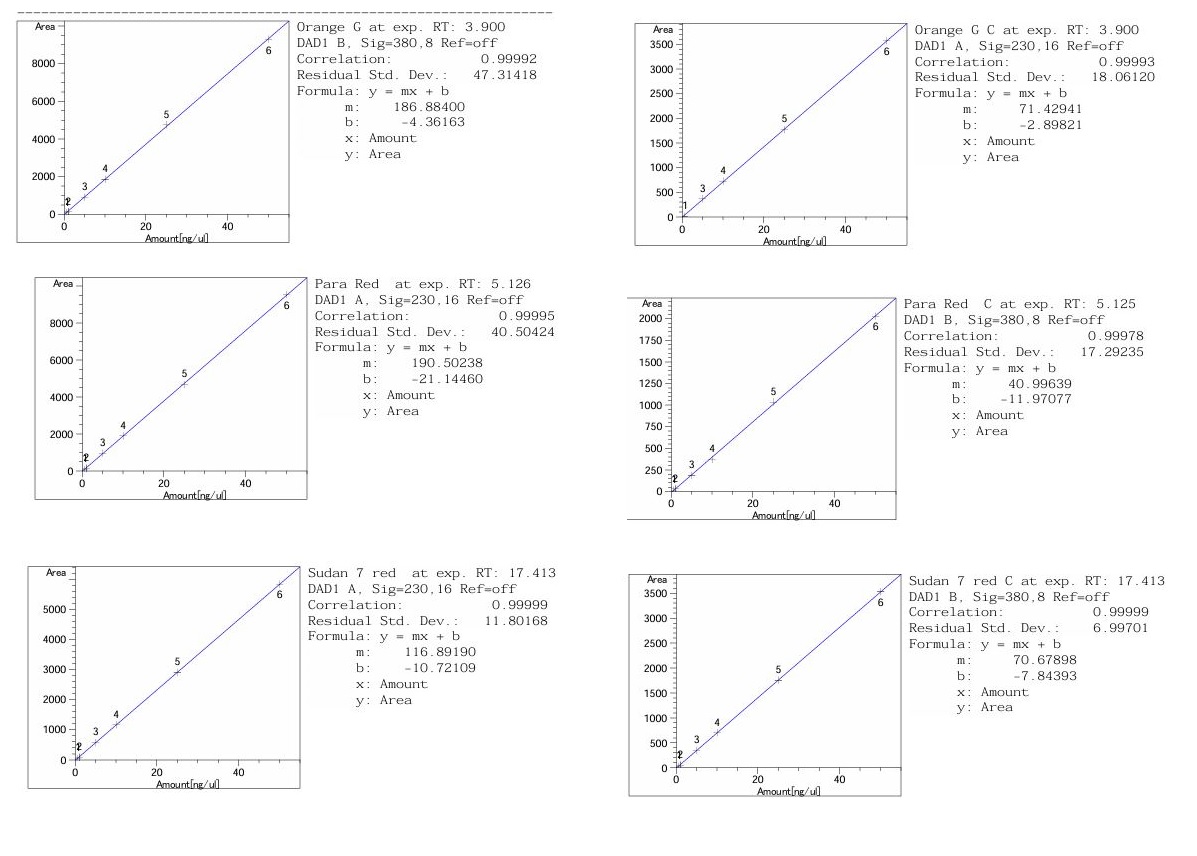
(a)**

**(b)**
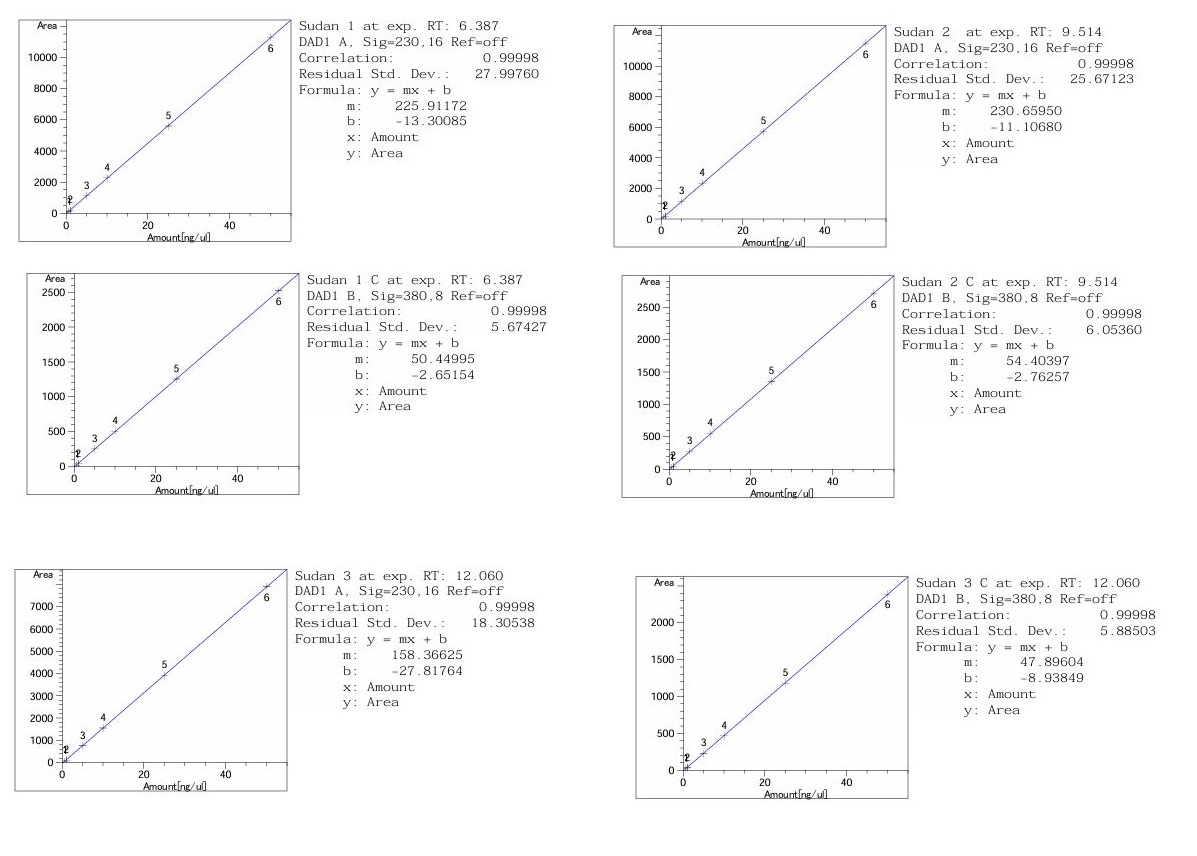


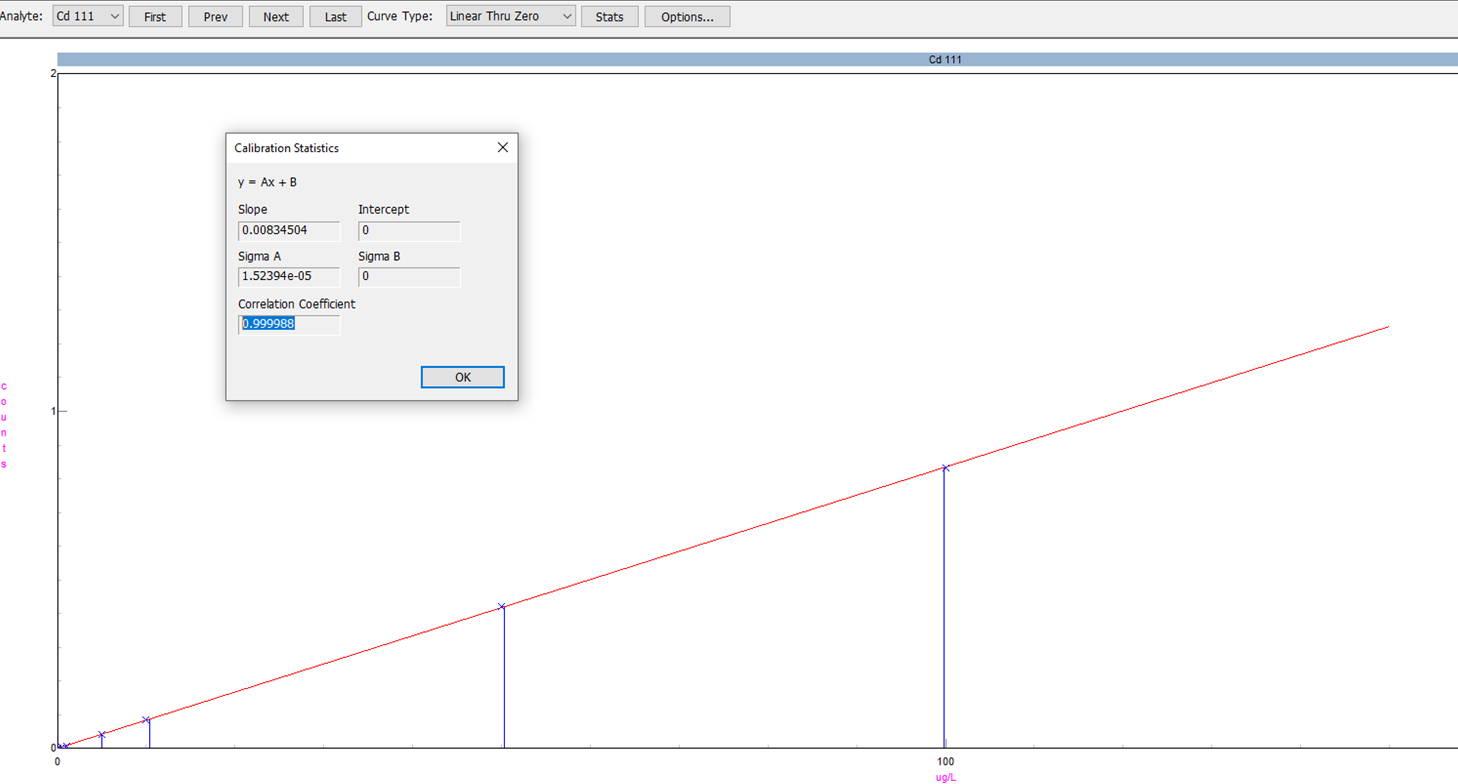
 **(C)**


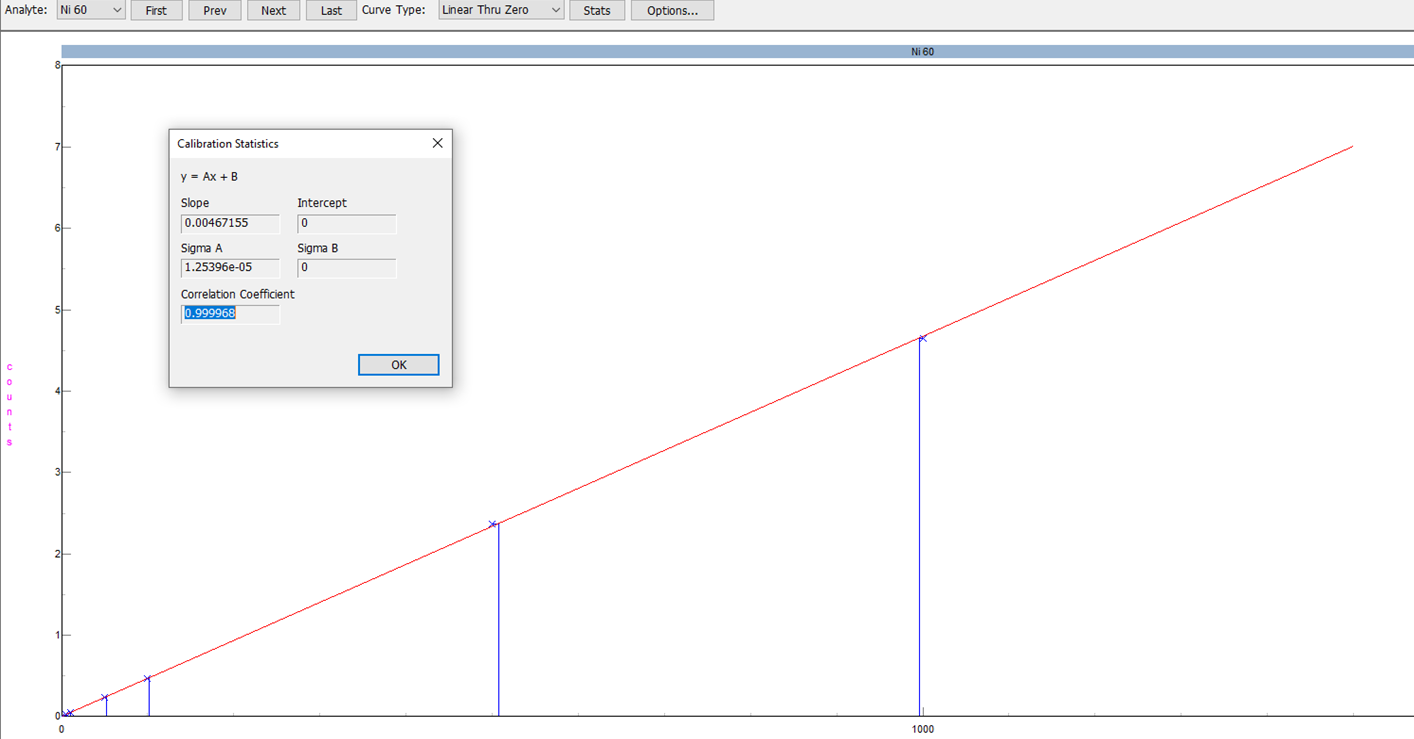


**(D)**


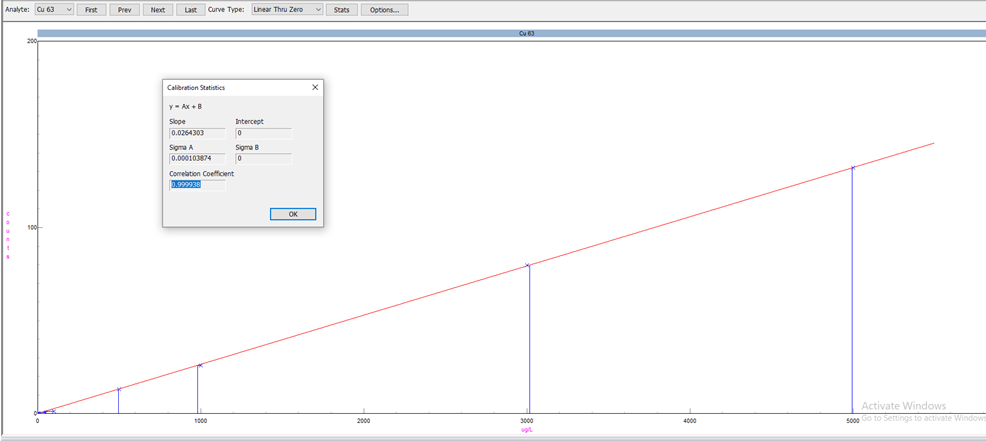


**(E)**

**(F)**

**(G)**

**(H)**

**(I)**

**(J)**

**(K)**

**(L)**

**Figure S1. Calibration curves (A)** Orange G, Sudan 7b, and Para-Red**, (B)** SD Ι, SD Π, and SD Ш**, (C)** Cadmium **, (D)** Nickle**, (E)** Copper**, (F)** Chlorpyrifos**, (G)** Chlorphenapy**r, (H)** Daizinon**, (I)** Malathon**, (J)** Pendimethalin**, (K)** Profenofos**,** and **(L)** Tebuconazole**.**
